# The characteristics of white dot syndromes following COVID-19 Vaccines: a systematic review

**DOI:** 10.1007/s10792-024-03119-4

**Published:** 2024-04-23

**Authors:** Hashem Abu Serhan, Husam Abu Suilik, Amr K. Hassan, Jehad Feras AlSamhori, Abdul Rhman Hassan, Abdelmonem Siddiq, Nagi Ahmed, Ayman G. Elnahry

**Affiliations:** 1 Department of Ophthalmology, Hamad Medical Corporations, Al Sadd, Al Rayyan St., PO: 3050, Doha, Qatar; 2 Faculty of Medicine, Hashemite University, Zarqa, Jordan; 3 Gavin Herbert Eye Institute, University of California, Irvine, CA USA; 4 School of Medicine, University of Jordan, Amman, Jordan; 5 Department of Ophthalmology, Visual and Anatomical Sciences, Wayne State University School of Medicine, Detroit, MI USA; 6 Faculty of Pharmacy, Mansoura University, Mansoura, Egypt; 7 Bascom Palmer Eye Institute, University of Miami, Miami, FL USA; 8 Department of Ophthalmology, Faculty of Medicine, Cairo University, Cairo, Egypt

**Keywords:** COVID-19, SARS-CoV-2, Chorioretinopathy, Multiple evanescent white dot syndrome, Ophthalmology

## Abstract

**Purpose:**

To review all studies reporting the onset of white dot syndromes following COVID-19 vaccines.

**Methods:**

Our protocol was registered prospectively on PROSPERO [registration number: CRD42023426012]. We searched five different databases including PubMed, Scopus, Web of Science, Google Scholar, and Science Direct up to May 2023. All the studies that reported the occurrence of white dot syndrome following COVID-19 vaccines were included. All statistical tests were conducted with a 95% confidence interval and a 5% error margin. A *p* value of less than 0.05 was considered statistically significant. The methodological quality of included studies was performed using the IHE Quality Appraisal Checklist for Case Series studies and JBI Critical Appraisal Checklist for Case Reports.

**Results:**

Fifty studies involving seventy-one subjects were included. Multiple evanescent white dot syndrome (MEWDS) was the most common disease (n = 25, 35.2% %), followed by acute macular neuroretinopathy (AMN) (n = 22, 31.0%) and acute posterior multifocal placoid pigment epitheliopathy (APMPPE) (n = 4, 5.6%). They were mostly unilateral (n = 50, 70.4%). The presenting symptoms were blurred vision (n = 26, 36.6%), paracentral scotoma (n = 19, 26.8%), visual field disturbance, and photopsia (n = 7, 9.9%). The mean duration for follow-up was 10.15 ± 14.04 weeks. Nineteen subjects (29.69%) received steroids with improvement reported in 68.4%. Eleven subjects (17.19%) were managed by observation only with reported full recovery and improvement.

**Conclusion:**

White dot syndromes are very rare entities. Our findings highlight a possible association between COVID-19 vaccines and the occurrence of white dot syndromes. However, larger studies with good quality should be implemented to confirm these findings.

**Supplementary Information:**

The online version contains supplementary material available at 10.1007/s10792-024-03119-4.

## Introduction

The severe acute respiratory syndrome coronavirus type 2 (SARS-CoV-2) pandemic has significant health consequences. Seven million fatalities have been reported from the 768 million confirmed cases of COVID-19 worldwide [1]. Nearly all the body's organs may be affected by the illness, which has the potential to cause respiratory issues ranging from those with no symptoms to those that are seriously uncomfortable or even fatal [2]. To control the COVID-19 pandemic, reliable and safe vaccines are crucial [3, 4]. As of the time of this writing, More than 13.4 billion doses of vaccines have been distributed [1] including messenger RNA vaccines (Pfizer-BioNTech and Moderna), inactivated vaccines (Sinopharm, Bharat Biotech, Sinovac), vector vaccines (Johnson & Johnson, AstraZeneca, Sputnik V), and protein subunit vaccinations (Novavax) [5]. Even though these vaccines were effective in halting the disease’s spread and lowering the incidence of severe forms of SARS-CoV-2, many potential adverse events were reported worldwide. [6, 7]

Until now, numerous unfavorable side effects, particularly those affecting the eyes, were reported [8]. For instance, some studies have reported that the Pfizer-BioNTech vaccine may cause about eye swelling, ocular hyperemia, conjunctivitis, blurred vision, uveitis, and visual impairment [6, 9]. Pfizer-BioNTech and AstraZeneca vaccines have been reported to result in ocular vascular events including vitreous hemorrhage, central/branch retinal vein occlusion, and ischemic optic neuropathy [10]. Mahendradas et al. [1] have reported anterior uveitis, intermediate uveitis, posterior uveitis, panuveitis, episcleritis, scleritis, sclerouveitis, sclerokeratouveitis, and keratouveitis following COVID-19 vaccines in their tertiary center.

White Dot Syndromes (WDS) are rare diseases of chorioretinopathy that have an annual incidence of 0.45 per 100,000 per year and typically affect young, healthy adults [11]. It has been reported that WDS can happen after vaccination for a number of diseases, including influenza, hepatitis B, polio, human papillomavirus (HPV), measles, mumps, rubella, and COVID-19 [12–18]. In fact, there growing evidence linking COVID-19 vaccinations and subtypes of WDS. For example, mRNA-1273 COVID-19 vaccine (Moderna) is associated with multiple evanescent white dot syndrome (MEWDS), and acute zonal occult outer retinopathy (AZOOR).[18, 19] In addition, acute macular neuroretinopathy (AMN), acute posterior multifocal placoid pigment epitheliopathy (APMPPE), and AZOOR have all been linked to the Pfizer-BioNTech vaccine administration. [17, 20–23] Furthermore, Medigen Vaccine Biologics Corporation (MVC) and Sinovac have been reported to cause MEWDS. [24, 25] Oxford-AstraZeneca, Sinopharm, and Johnson & Johnson also were reported to cause AMN [26–29].

The development of WDS following COVID-19 vaccination should therefore receive more attention. The importance of this recent possible association has been emphasized in several recent studies that have been published in this field but are solely based on case reports. As a result, we conducted a systematic review with a focus on the data regarding the vaccines (type, dose, duration), the patient's characteristics (sociodemographic and clinical), and the outcomes of the disease (origin, type, location, presentation, management, and outcomes) to summarize the current evidence on COVID-19 vaccine-associated WDS. This is the first systematic review that, to our knowledge, addresses WDS that develops following COVID-19 vaccination.

## Materials and methods

### Study protocol and database search

This research was carried out in accordance with the Preferred Reporting for Systematic Review and Meta-Analysis (PRISMA) recommendations [30, 31].The study adhered to the tenets of the Declaration of Helsinki and the necessity for institutional review board (IRB) approval was not required since it did not involve human subjects. In May 2023, our protocol was registered prospectively on PROSPERO [registration number: CRD42023426012].

Meanwhile, on May 25, 2023, we searched five electronic databases [PubMed, Scopus, Web of Science, ScienceDirect, and Google Scholar] to retrieve all studies that reported the onset of any type of WDS following COVID-19 vaccines within 8 weeks using the following keywords: (Pfizer-BioNTech OR BTN162b2 OR Sinopharm OR Sinovac OR Moderna OR AstraZeneca OR ChAdOx1 OR AZD1222 OR Janssen OR “Johnson & Johnson” OR Novavax OR CoronaVac OR Covaxin OR Convidecia OR Sputnik OR Zifivax OR Corbevax OR COVIran OR SCB-2019 OR vaccin* OR “COVID-19 Vaccines”[Mesh]) AND ((“white dot syndrome” OR “multiple evanescent white dot syndrome” OR “acute idiopathic blind spot enlargement syndrome” OR “punctate inner chorioretinop*” OR “acute macular neuroretinopath*” OR “acute posterior multifocal placoid pigment epitheliopathy” OR “diffuse subretinal fibrosis uveitis” OR “serpiginous choroid*” OR “birdshot chorioretinopathy*” OR “acute zonal occult outer retinopathy”))). Medical Subject Headings (MeSH) terms were also added whenever applicable to retrieve all relevant studies based on their indexed terms in included databases. In addition, only the first 200 records from Google Scholar were retrieved and screened as per the recent recommendations [30]. Noteworthy, an updated database search was carried out just before the analysis to include any newly published studies before the official synthesis of collected data which didn’t yield any new results.

Furthermore, after finishing the screening process, we conducted a manual search of references to identify any relevant studies that we could not identify through the original database search. This search was conducted by (1) searching similar articles of the finally included articles in our review through the “similar articles” option on PubMed, (2) searching the reference list of finally included articles in our review, and (3) searching through Google with the keywords used in the original database search.

### Eligibility criteria

We included all original research papers that reported the onset of any type of WDSs following any type of COVID-19 vaccine within 8 weeks of vaccine administration following the PICO framework (Population: subjects with any type of WDS, Intervention: COVID-19 vaccines, Comparator: none, Outcome: presentation, management, and prognostic factors. We included all of the following study designs: randomized controlled clinical trials (RCT), retrospective and observational studies, case series, and case reports. Of note, studies were included regardless of the language of publication. Meanwhile, studies were excluded if they were (1) non-original research (i.e., reviews, commentaries, guidelines, editorials, correspondence, letters to editors, opinions etc.), (2) unavailable full-texts, (3) duplicated records or records with overlapping datasets, (4) studies reporting WDSs following SARS-CoV-2 infection (5) studies with irrelevant data (lack of primary outcome data) (6) studies reporting WDSs following COVID-19 vaccines within > 8 weeks.

### Screening and study selection

Retrieved records from the database search were exported into EndNote software for duplicate removal before the beginning of the screening phase. Records were then imported into an Excel (Microsoft, USA) sheet for screening. The screening was divided into two steps: title and abstract screening followed by full-text screening. The full texts of eligible articles were then retrieved for screening before being finally included in the review. Both steps were carried out by three reviewers [AKH, ARH, AS]. Any differences between reviewers were solved through group discussions, and the senior authors [HAS, AGE] were consulted if reviewers could not reach an agreement.

### Data extraction and assessment of methodological quality and risk of bias

The data extraction was performed by three reviewers [AKH, ARH, AS] through a data extraction sheet that was formatted through Excel (Microsoft, USA). This sheet consisted of five parts. The first part included the baseline characteristics of included studies [title, authors’ names, year of publication, country, and study design] and patients as well [sample size, age, and gender]. The second part included data on the reported WDS events (name, type, number, and laterality [right or left eye]), COVID-19 vaccine (type, number of doses, time from vaccine administration to symptoms onset, and SARS-CoV-2 infection status). The third part summarized the medical history of the reported cases with WDS events (i.e., systemic diseases, cardiovascular diseases, cerebrovascular diseases, immunological diseases, history of eye trauma, previous eye diseases, and previous ocular surgeries). The fourth part included a thorough assessment of the reported event in terms of presenting symptoms, diagnostic methods, examination findings, initial and final best-corrected visual acuity (BCVA), investigations (blood and eye investigations), management (either medical or surgical), the follow-up period, and management outcomes and associated complications if present. The fifth part included the quality assessment of the included studies. Methodological quality and risk of bias were assessed using the IHE Quality Appraisal Checklist for Case Series studies [32] and the JBI Critical Appraisal Checklist for Case Reports. [33]

### Data synthesis

No modifications have been made to the pre-defined analysis plan in the study protocol. We performed qualitative analysis after organizing the acquired data. Qualitative analysis was done using the Statistical Package for Social Sciences (SPSS) version 27 (IBM SPSS Corp, SPSS Statistics ver. 27, USA). Descriptive analysis was used to display categorical variables as percentages and frequencies while presenting numerical variables as a mean and standard deviation. We tried to run time-to-event analysis for better understanding of relation of theWDS to the vaccines. The significance of the data was determined using a categorical Chi-square test. All statistical tests were conducted with a 95% confidence interval and a 5% error margin. A p-value of less than 0.05 was considered statistically significant. Visual acuity (VA) was commonly reported as an Early Treatment Diabetic Retinopathy Study letter scores. We standardized VA scores using the minimum angle of resolution (logMAR) chart scores, the score was converted to logMAR scores using Gregori et al. method. [34]

## Results

**Fig. 1:**
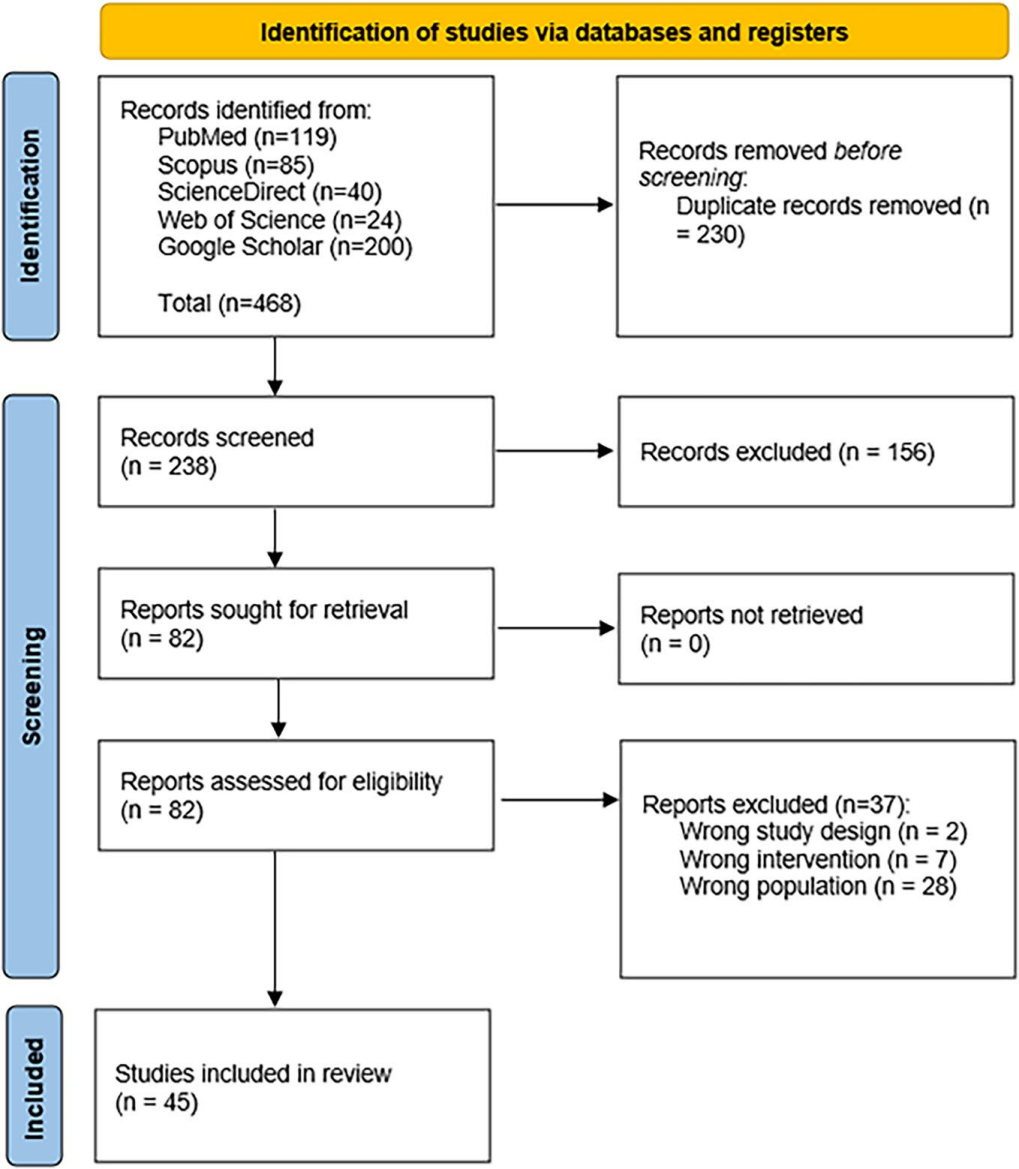
Shows PRISMA chart for selection of included articles

The search strategy retrieved 240 references and forty-five studies were included. (Fig. 1) Thirty-two were case reports (71.11%) [12, 18, 20, 24–26, 35–64] and 13 were case series (31.11%). [17, 65–76] Sixty-four patients were included. Their age average was 32.60 ± 13.76 (mean ± SD). Female patients were fifty-one (80.95%) and males were twelve (19.05%) and one case didn’t report the gender. The studies originated from twenty-two countries, Europe (n = 15, 33.33%), and USA (n = 10, 22.22%) were the most. (Table 1) The quality assessment and overall appraisal of the included articles is shown in Supplementary Table 1 and 2.

**Table 1:** Demographic description of included articles and related ophthalmological findings

Author (ID)	Study design	Country	Age	Gender	Past ophthalmic Hx	Comorbidities	Visual acuity (LogMAR)
Alhabshan et al. [35]	Case report	USA	71	Female	Retinal tear OS treated with laser a few years ago	Hypercholesterolemia	OD 0.09 OS 0.2
Book et al. [65]	Case series	India	20	Female	Healed choroiditis scar OS & Active TB choroiditis OD treated with intravitreal dexamethasone implant, 4 drug Anti TB regimen	Positive tuberculin test	1.8 OD
Book et al. [65]	Case series	India	26	Male	SLC 2 years ago treated with oral steroids and 4 anti-TB drugs	NA	0 OU
Capuano et al. [20]	Case report	Turkey	45	Female	NA	NA	0.69 OD 0 OS
Conrady et al. ([26]	Case report	India	22	Female	NA	NA	0 OD 0.2 OS
David et al. [36]	Case report	USA	40	Female	None	Type 2 DM, Hyperlipidemia, Hypothyroidism	OD 0 OS 1.8
De Salvo et al. [37]	Case report	USA	64	Female	NA	Hypertension, deep vein thrombosis on dabigatran, and a 30-pack year smoking history	1 OU
El Matri et al. [38]	Case report	Norway	27	Female	NA	OCP	0 OU
Fekri et al. [66]	Case series	Italy	53	Male	NA	NA	0.09 OS
Fekri et al. [66]	Case series	Italy	18	Female	NA	NA	0.51 OD
Fekri et al. [66]	Case series	Italy	48	Male	NA	NA	1.3 OD
Fekri et al. [66]	Case series	Italy	25	Female	NA	OCP	0 OU
Fischer et al. [39]	Case report	Germany	21	Female	NA	Levonorgestrel-Ethinyl Estradiol Oral	0 OU
Gabrielle et al. [17]	Case series	Canada	28	Female	NA	Oral HSV	0.47 OD
Gabrielle et al. [17]	Case series	Canada	27	Male	NA	NA	0 OU
Gabrielle et al. [17]	Case series	Canada	26	Female	NA	NA	0 OS
Hawley et al. [40]	Case report	UK	58	Male	NA	Palmoplantar Pustular Psoriasis after 1st dose of vaccine	1 OD 2.3 OS
Patel et al. [41]	Case report	Germany	23	Female	NA	Juvenile idiopathic arthritis & associated recurrent iritis, OCP	0 OU
Rennie et al. [42]	Case report	Iran	18	Female	NA	NA	0 OU
Vinzamuri et al. [67]	Case series	Austria	19	Female	NA	Asthma, OCP	0 OU
Vinzamuri et al. [67]	Case series	Austria	31	Female	NA	OCP	0 OU
Virgo et al. [43]	Case report	France	25	Female	NA	COC	0 OU
Xu et al. ([68]	Case series	France	40	Male	NA	NA	0 OD 0.09 OS
Xu et al. [68]	Case series	France	23	Female	NA	NA	0 OU
Zaheer et al. [69]	Case series	Switzerland	21	Female	NA	NA	0 OU
Fowler et al. [44]	Case report	India	34	Male	NA	NA	0.77 OD, 0 OS
Mélanie H. et al. [45]	Case report	Canada	41	Male	Mild myopia − 2.75/ − 2.5 diopters	NA	0.69 OD, 0.09 OS
Ahmed et al. [46]	Case report	Japan	30	Female	NA	NA	1 OU
Ahmed et al. [70]	Case Series	Japan	33	Female	NA	HTN, Alport syndrome, ESRD	1 OS
Azar et al. [12]	Case Report	USA	17	Male	NA	NA	0 OU
Azar et al. [88]	Case Series	Netherlands	42	Female	NA	Oral Contraceptives for 10 years	NA
Azar et al. [88]	Case Series	Netherlands	40	Female	NA	NA	NA
Cuadros Sánchezet al [47]	Case Report	South Korea	83	Female	NA	HTN, Hyperlipidemia on anti HTN & Lipid lowering agents	2.3 OD 0.2 OS
Cunha Ângelo et al. [71]	Case Series	China	79	Female	NA	NA	0.6 OS
Cunningham et al. [72]	Case Series	China	18	Female	NA	NA	0.9 OU
Cunningham et al. [72]	Case Series	China	49	Male	NA	NA	0.7 OD 0.1 OS
Cunningham et al. [72]	Case Series	China	25	Female	NA	NA	0.6 OD 1 OS
Cunningham et al. [72]	Case Series	China	42	Female	NA	NA	1.8 OD 0.4 OS
Cunningham et al. [72]	Case Series	China	24	Male	NA	NA	0.8 OD 0.2 OS
Cunningham et al. [72]	Case Series	China	38	Female	NA	NA	1 OD 0.4 OS
Cunningham et al. [72]	Case Series	China	37	Female	NA	NA	0.2 OD 0.5 OS
Cunningham et al. [72]	Case Series	China	31	Female	NA	NA	NA
Cunningham et al. [72]	Case Series	China	32	Female	NA	NA	0.4 OD 0.5 OS
Cunningham et al. [72]	Case Series	China	21	Female	NA	NA	0.15 OD 1 OS
Da Silva et al. [24]	Case Report	Taiwan	36	Female	High myopia, spherical equivalent refraction was − 9.75D and − 7.5D OD & OS	NA	0.09 OU
Detrick et al. [73]	Case Series	UK	22	Female	NA	NA	0 OU
Diafas Et al [48]	Case Report	Ireland	40	Female	NA	NA	0.47 OD, 1.77 OS
Dumitru [49]	Case Report	France	21	Female	NA	NA	0
Fine et al. [50]	Case Report	India	14	Female	NA	NA	1.8 OS, 0 OD
Gliem et al. [51]	Case Report	France	30	Female	Bilateral operated crystalline lens subluxation in childhood. Four months ago, she underwent a left trans-scleral suture less intraocular lens fixation	NA	1 OD, 0 OS
Gross et al. [52]	Case Report	Japan	31	Female	NA	NA	0.69 OD, 1 OS
Haseeb et al. [53]	Case Report	USA	26	Female	NA	NA	0 OU
Helal et al. [54]	Case Report	USA, UAE	NA	NA	Central serous chorioretinopathy with chronic serous pigment epithelial detachment in the OS	NA	1.3 OU
Khochtali et al. [74]	Case Series	Israel	39	Male	NA	NA	0.2 OS
Khochtali et al. [74]	Case Series	Israel	28	Female	NA	NA	0.2 OS
Kim et al. [55]	Case Report	Canada	39	Male	NA	NA	0 OU
Li et al. [56]	Case Report	USA	21	Female	NA	NA	0.69 OD, 0 OS
McMichael et al. [57]	Case Report	India	25	Female	NA	Microcytic hypochromic anemia, and during electrophoresis, she was detected to have a β thalassemia trait	0 OU
Ng et al. [58]	Case Report	South Korea	33	Female	NA	NA	0.3 OD
Papasavvas et al. [75]	Case Series	Australia	15	Male	NA	NA	0.69 OD, 0 OS
Papasavvas et al. [75]	Case Series	Australia	21	Female	NA	NA	0.47 OD, 0 OS
Prieto-Peña et al. [18]	Case Report	USA	31	Female	Significant for myopia, and she was status-post myopic LASIK surgery in OU in 2019	NA	1 OD, 0.39 OS
Roy et al. [25]	Case Report	Brazil	38	Female	NA	NA	1.3 OD, 0 OS
Sasajima et al. [59]	Case Report	USA	20	Female	NA	NA	0 OU
Shah et al. [60]	Case Report	India	34	Male	NA	NA	0 OU
Sriwijitalai et al. [76]	Case Series	Taiwan	32	Female	NA	NA	0.3 OD
Sutandi et al. [61]	Case Report	USA	17	Female	NA	Migraine	NA
Tomkins-Netzer et al. [62]	Case Report	China	40	Female	Previous episode of MEWDS	Had experienced an episode of transient hypertension (193/ 107 mmHg)	0.69 OS, 0 OD
Wang et al. [63]	Case Report	Japan	67	Female	NA	NA	0.69 OD, 0 OS
Yang et al. [64]	Case Report	UK	22	Female	NA	NA	0 OU

Author (ID)	OCT	FA	Other investigations	Covid Vaccination	Number of doses	Duration from taking the vaccine till onset of symptoms (days)	Any remarkable complication of vaccination	Author (ID)
Alhabshan et al. [35]	Multiple small subretinal hyper-reflective lesions in the macula that were more concentrated nasally with disruption of the EZ	Hyperfluorescence of the retinal lesions in a characteristic wreath pattern with no evidence of leakage	Fundoscopy: multiple faint outer retinal lesions in the peripapillary and peri-macular areas extending to the periphery that were white-yellow, myopic fundus, PVD. FAF: Hyperautofluorescence. VF: Enlarged blind field	mRNA-1273 vaccine	2	3	Mild malaise and soreness at the site of injection that lasted for 1 day	Alhabshan et al. [35]
Book et al. [65]	NA	NA	Fundoscopy: OD Active choroiditis, FAF: hyperautofluorescence corresponding to the active choroiditis lesion in the OD while hypoautoflourescent choroiditis scar was present OS	Covishield vaccine	1	14	NA	Book et al. [65]
Book et al. [65]	NA	NA	Anterior segment examination of the OD revealed 1 + cellular reaction while fundus examination showed 2 + vitritis and active choroiditis lesions in macular area with healed lesions in the midperiphery. The anterior and posterior segment examination of OS was normal. FAF: hyperautofluorescence corresponding to the active choroiditis lesion	Covishield vaccine	1	42	NA	Book et al. [65]
Capuano et al. [20]	Subretinal fluid together with an appearance of bacillary layer detachment at the right macula, choriocapillaris slab of the right fundus showed scattered flow deficit areas, OS Normal	Scattered initially hypo and then hyperfluorescent scattered spots at the posterior pole in the OD, OS Normal	Fundus examination of the OD revealed multiple discrete yellow–white placoid lesions at the level of deep retinal layers throughout the posterior pole, OS Normal but Two weeks after the initial eye examination, similar multiple placoid lesions were observed in her OS	mRNA	1	7	NA	Capuano et al. [20]
Conrady et al. ([26]	Disruptions in the outer retina involving the EZ, IZ, and ELM. Hyperreflective dots were seen in the ONL	NA	The Fundus examination of the OS showed a mild vitreous haze. Multifocal yellowish-white, deep retinal, small and larger dots were seen in the posterior pole and extended beyond the arcades. FAF showed multiple scattered hyperautofluorescent spots: OS revealed a generalized reduction and paracentral islands of sensitivity loss	Covishield	2	1	Low-grade fever and pain at the injection site following the first as well as second dose of the vaccine	Conrady et al. ([26]
David et al. [36]	OS Retinal thickening and edema of outer retinal layers more nasally, optic nerve extensive thickening of RNFL		Slit lamp (OS Flare, OD + 1 vitreous cells, OU trace cell, + 2 nuclear sclerotic cataract) Fundoscopy (Multiple non coalescing creamy colored placoid lesions throughout posterior pole, OS severe papillitis and superior disc hemorrhages with surrounding retinal edema), Brain MRI (contrast enhancement within posterior aspect of left globe at optic nerve sheath insertion)	Pfizer-BioNTech	1	14	NA	David et al. [36]
De Salvo et al. [37]	Outer nuclear layer thinning and ellipsoid zone disruption OU	Normal	The dilated fundus exam showed subtle, bilateral, pigmentary changes in the central maculae. Near-infrared reflectance revealed hyporeflective lesions in the maculae OU. OCT-angiography showed flow voids in the deep capillary plexus and choriocapillaris OU. FAF showed subtle, granular hypoautofluorescence in the foveal region of OU	Moderna mRNA	1	1	New onset malaise, vomiting and diarrhea requiring hospital admission	De Salvo et al. [37]
El Matri et al. [38]	Slight hyperreflectivity of the outer nuclear and plexiform layers and disruption of the ellipsoid zone	NA	The threshold perimetry of the OS showed a modest paracentral scotoma in the upper temporal quadrant. Fundoscopy of the OS revealed a delicate teardrop-shaped macular lesion nasally to the fovea. OCT angiography indicated subtle dropout in the deep capillary plexus corresponding to the lesion	AstraZeneca vaccine	1	2	The same day she developed flu-like symptoms. These resolved 2 days later	El Matri et al. [38]
Fekri et al. [66]	NA	NA	NA	BNT162b2	2	28	NA	Fekri et al. [66]
Fekri et al. [66]	NA	NA	NA	BNT162b2	1	4	NA	Fekri et al. [66]
Fekri et al. [66]	NA	NA	NA	BNT162b2	1	7	NA	Fekri et al. [66]
Fekri et al. [66]	NA	NA	NA	ChAdOx1 nCoV-19	1	2	NA	Fekri et al. [66]
Fischer et al. [39]	Outer plexiform layer thickening and discontinuity of the photoreceptor inner-segment ellipsoid band	NA	Bilateral circumscribed paracentral dark lesions that were easily visible on in-framed reflectance imaging. Microperimetry demonstrated bilateral scotomas corresponding to these lesions	Vaxzervria	1	3	NA	Fischer et al. [39]
Gabrielle et al. [17]	Disruption of the outer retina with hyperreflective projections extending to the outer nuclear layer	Early hyperfluorescent lesions in early frames with late staining of these lesions in a wreathlike configuration	Fundoscopy: multiple small deep grey, white lesions throughout the posterior pole. FAF widespread punctiform hyperautofluorescent foci corresponding to the lesions on examination	Moderna	2	14	NA	Gabrielle et al. [17]
Gabrielle et al. [17]	NA	NA	Blunting of the foveal reflex and multiple deep greyish posterior- pole lesions	Pfizer-BioNTech-BioNTech vaccine	1	22	NA	Gabrielle et al. [17]
Gabrielle et al. [17]	Normal	Early hyperfluorescent lesions in the early frames with late staining of lesions	Fundoscopy: pigmentary punctiform changes in the posterior pole	Pfizer-BioNTech-BioNTech	2	5	NA	Gabrielle et al. [17]
Hawley et al. [40]	Alterations in the outer retinal layers with disruption of the interdigitation zone, inner segment-outer segment junction and the external limiting membrane with evidence of bacillary layer detachment, sub-foveal RPE proliferation as well as subretinal and intraretinal fluid in OU	NA	Fundus examination revealed bilateral well-delineated whitish plaque-like macular lesions involving the fovea. FAF the lesions demonstrated mottled hyperautofluorescence with intervening spots of hypoautofluorescence, more speckled in the OS. FAF early hypofluorescence corresponding to the lesions seen clinically, with punctate areas of hyperfluorescence and late staining	AstraZeneca	2	12	Palmoplantar Pustular Psoriasis after 1st dose of vaccine	Hawley et al. [40]
Patel et al. [41]	Corresponding to fundoscopy images hyperreflective lesions of the outer retina with a thickened OPL, a thinning of the ONL, and a disruption of the ELM, the EZ and the interdigitation zone (IZ)	NA	Fundoscopy: subtle brownish rimmed lesion parafoveal in the OD and a bigger blurred lesion nasal to the macula in the OS. IR two distinct hyporeflective lesions located parafoveal superior-nasally in the OS and a smaller grayish area inferior-nasally of the macula in the OD, OCTA: no reduced flow in the superficial and deep capillary plexus. However, we noted a subtle flow void in the choriocapillaris	AstraZeneca	NA	NA	Headache and cervical pain on the first day after vaccination	Patel et al. [41]
Rennie et al. [42]	OPL thickening, ONL thinning, and disruption of the ellipsoid zone in areas corresponding to the lesions (OU)	No vascular pathology	Fundoscopy: hypopigmented perifoveal areas. VF: paracentral scotomas in OU. IR: two perifoveal hyporeflective lesions OD. One supertemporal (wedge-shaped) and the other inferior and inferotemporal to the fovea. And a diffuse perifoveal semi-circular hyperreflective lesion OS extending from the inferonasal axis to the supertemporal axis. OCTA: no flow abnormality at superficial/deep retinal vasculature or choriocapillaris	Sinopharm	NA	5	A day later, she experienced flu-like symptoms Including headache, fatigue, fever, and chills	Rennie et al. [42]
Vinzamuri et al. [67]	Area of parafoveal hyperreflective change in the OPL and outer nuclear layer ONL with disruption of the EZ and IZ. These lesions correlated with a wedge-shaped hypo reflective area on the near-infrared images	NA	Fundoscopy: large, opaque-appearing parafoveal wedge-shaped areas. VF: Paracentral scotoma. merged: Normal	AstraZeneca	NA	1	Flu-like symptoms with headache and temperature up to 39°C, which resolved by itself during the first 48 h	Vinzamuri et al. [67]
Vinzamuri et al. [67]	Parafoveal hyperreflective change in the OPL and ONL with disruption of the EZ and IZ of the photoreceptor layers on OU. These lesions correlated with a wedge-shaped hypo reflective area on the near-infrared images	NA	Fundoscopy: OD revealed a small opaque-appearing area superior to the fovea and was unremarkable for the OS. VF: scotoma inferior to the fovea on the OD with diffuse, unspecific reduction of the Bebié curve on the OS. merged: diminished parafoveal amplitudes on the OD. OCTA: altered flow in the DCP of the affected areas	AstraZeneca	NA	2	Flu-like symptoms with headache and temperature up to 39°C, which resolved by itself during the first 24 h	Vinzamuri et al. [67]
Virgo et al. [43]	Multiple bilateral localized hyperreflective macular lesions of the outer plexiform layer and Henle layer associated with thinning of the outer nuclear layer, as well as localized disruption of the ellipsoid zone (EZ) and interdigitation zone and an attenuated external limiting membrane	NA	Color Fundus photography and autofluorescence imaging were unremarkable in OU. NIR: multiple classic hyporeflective parafoveal wedge-shaped areas. OCTA does not show any vascular abnormalities in either eye, notably in the deep retinal capillary plexus, choriocapillaris and choroid. VF: Normal. Dye fundus angiography was unremarkable with normal transit and no sign of posterior uveitis	AstraZeneca	1	1	NA	Virgo et al. [43]
Xu et al. ([68]	Hyperreflective dots in the outer nuclear layer (ONL) associated to a disruption of the EZ	NA	Funduscopic examination OS showed multiple granular white dots with an aspect of foveal granularity. FAF: numerous hyperautofluorescent lesions that were underappreciated clinically. FFA: early bilateral granular hyperfluorescence with staining of those lesions at the early phase. ICG-A was normal in the early and intermediate phase, but also showed numerous hypofluorescent dots that were predominant in the mid-periphery	mRNA vaccine	1	7	NA	Xu et al. ([68]
Xu et al. [68]	Disruption of the outer retinal, specifically in the ellipsoid zone (EZ) with a granular appearance	NA	Fundus examination OS showed altered macular reflex with an aspect of foveal granularity. FAF numerous hyperautofluorescent spots that were also underappreciated clinically. FFA early bilateral granular hyperfluorescence that correlates with the granularity seen on dilated fundus examination, with staining of those lesions at the early phase that decreased at the intermediate phase and the late phase. ICGA normal in early and intermediate phase but has numerous hypofluorescent dots predominantly in the midperiphery and around the optic disc during the late phase	mRNA	1	10	NA	Xu et al. [68]
Zaheer et al. [69]	Parafoveal lesions OU, alterations in outer retinal layers OS > OD	NA	Perimetry: small, round, central scotomas OU	Vaxzervria	1	3	NA	Zaheer et al. [69]
Fowler et al. [44]	Massive subretinal fluid at the macula in RE with a smaller serous detachment inferonasal to the optic disc; OS had a small pocket of subretinal fluid temporal to the optic disc not involving the macular center	NA	Fundus examination revealed bilateral multiple yellowish, round to oval lesions at the level of the Choroid clustered at the macula and optic nerve extending into the mid periphery with serous detachments of the retina at multiple locations in OU. B-scan ultrasonography revealed significant choroidal thickening in OU. The choroidal thickness (CT) in the RE was 4 times more than normal and LE was 2 times more than normal	Covishield	2	9	Twenty-four hours post-vaccination he had severe frontal Headache, ocular pain, mild pain at the injection site and generalized myalgia for the first two days	Fowler et al. [44]
Mélanie H. et al. [45]	NA	Peripheral nonperfusion and vasculitis OU, as well as a peripapillary pigment epithelial detachment OD	Dilated fundus examination was normal OU without signs of macular edema, chorioretinitis, optic nerve edema, granulomas, or vascular sheathing.2 weeks later dilated fundus exam showed spillover of cells in the anterior vitreous with vascular sheathing OU and two yellowish infiltrates along the inferior arcade OD	Pfizer-BioNTech-BioNTech	1	2	NA	Mélanie H. et al. [45]
Ahmed et al. [46]	EZ disruption superior to fovea	Early hyperfluorescent spots, circumferentially distributed around fovea	Fundoscopy (Spread of spots OS > OD, vitreous cells OS > OD)	BNT162b2 mRNA vaccine	2	3	NA	Ahmed et al. [46]
Ahmed et al. [70]	Hyperreflective lesions affecting the outer plexiform and outer nuclear layers with disruption of the ellipsoid zone	NA	OCTA OS possible flow deficits in the deep capillary plexus	Pfizer-BioNTech-BioNTech	2	8	NA	Ahmed et al. [70]
Azar et al. [12]	Focal areas of disruption of the ellipsoid layer, outer retina, and retinal pigment epithelium	Early hypofluorescence and late staining of the placoid lesions in the OD	Fundoscopy: multiple flat, creamy white placoid subretinal lesions within the posterior pole and temporal midperipheral retina of the OD, FAF confirmed APMPPE demonstrated multiple hypoautofluorescent placoid lesions in the posterior pole and midperiphery with hyperautofluorescent borders. OCTA OD demonstrated hypoperfusion of the choriocapillaris in a placoid pattern that was more extensive than the clinically observed creamy-white lesions	Pfizer-BioNTech-BioNTech	1	14	NA	Azar et al. [12]
Azar et al. [88]	OD Subtle irregularities of outer nuclear & photoreceptor layers	NA	Fundoscopy (OD Faint brownish circle nasal to fovea) VF (Temporal scotoma)	Spikevax	2	45	NA	Azar et al. [88]
Azar et al. [88]	Single round spot with neuroretina changes	NA	Fundoscopy (Mild pigmentary changes nasal superior fovea) VF (Temporal inferior scotoma)	Spikevax	2	45	NA	Azar et al. [88]
Cuadros Sánchezet al [47]	Optic nerve head swelling in the OD and subretinal fluid and disruption of the photoreceptor layers in OU	Multiple focal leakages from the retinal vessels in OU	VF: generalized field defect in the OD and superior arcuate scotoma in the OS. MRI: subtle enhancement of the right optic nerve in the retrobulbar area, while MRI findings in the area from the optic nerve head to the chiasm in the OS were normal	Pfizer-BioNTech-BioNTech	2	2	Immediately developed malaise and limb pain. Two days later, the pain in her upper and lower extremities had decreased	Cuadros Sánchezet al [47]
Cunha Ângelo et al. [71]	NA	NA	NA	Pfizer-BioNTech-BioNTech	2	14	NA	Cunha Ângelo et al. [71]
Cunningham et al. [72]	NA	NA	NA	Inactivated COVID 19 vaccine	2	5	NA	Cunningham et al. [72]
Cunningham et al. [72]	NA	NA	NA	Inactivated COVID 19 vaccine	1	7	NA	Cunningham et al. [72]
Cunningham et al. [72]	NA	NA	NA	Inactivated COVID 19 vaccine	1	4	NA	Cunningham et al. [72]
Cunningham et al. [72]	NA	NA	NA	Inactivated COVID 19 vaccine	1	2	NA	Cunningham et al. [72]
Cunningham et al. [72]	NA	NA	NA	Inactivated COVID 19 vaccine	3	15	NA	Cunningham et al. [72]
Cunningham et al. [72]	NA	NA	NA	Inactivated COVID 19 vaccine	1	15	NA	Cunningham et al. [72]
Cunningham et al. [72]	NA	NA	NA	Inactivated COVID 19 vaccine	2	3	NA	Cunningham et al. [72]
Cunningham et al. [72]	NA	NA	NA	Inactivated COVID 19 vaccine	1	2	NA	Cunningham et al. [72]
Cunningham et al. [72]	NA	NA	NA	Inactivated COVID 19 vaccine	1	3	NA	Cunningham et al. [72]
Cunningham et al. [72]	NA	NA	NA	Inactivated COVID 19 vaccine	1	7	NA	Cunningham et al. [72]
Da Silva et al. [24]	Hypo reflective lesion in the ellipsoid zone of the perifoveal lesions in the OD	Early hyperfluorescence, and indocyanine green angiography (ICGA) captured late hypocyanescent spots	The color fundus imaging of the OD showed multiple whitish round spots in the posterior pole extending to the mid-periphery of the retina. VF: paracentral scotoma with mean deviation of 5.57 db in the OD. The paracentral scotoma was prominent in the inferior nasal area in the OD, which was corresponding to her flashing lights symptom and the white dots lesion in the fundus imaging	MVC COVID-19 Vaccination	1	2	Mild fatigue, and the highest recorded body temperature was 37.5° Celsius	Da Silva et al. [24]
Detrick et al. [73]	Suggestive of AMN in both cases	Unremarkable for both cases	Case 1: Blood tests: showed reduced neutrophil count and very mild thrombo- cytopenia (141 000/μL – reference values 150–450 000/μL)	Vaxzervria	1	NA	NA	Detrick et al. [73]
Diafas Et al [48]	Hyperreflectivity and disruption to the outer retinal layers from outer plexiform layer (OPL) to the RPE in both foveae	correlating well-circumscribed lesions with early dense hypofluoroescence, followed by late hyperfluoroescence	Serological investigations and a chest X-ray ruled out infectious, autoimmune, and inflammatory aetiologias while MRI of the brain ruled out central nervous system (CNS) vasculitis	Pfizer-BioNTech-BioNTech Manufacturing	2	NA	NA	Diafas Et al [48]
Dumitru [49]	Multifocal highly reflective lesions at the junction of the outer plexiform layer (OPL) and outer nuclear layers (ONL), with disruption of the underlying ellipsoid and interdigitation zones (EZ/IZ) consistent with AMN	Subtle macular hypoautofluorescence was observed on the left side	Initial laboratory work- up revealed inflammatory syndrome with high-level of C-Reactive protein (54 mg/L) and a leukopenia (2.6 G/L) Platelets were normal (278 G/L). Coagulation screening tests, including prothrombin time and activated partial thromboplastin time were normal. She was tested negative for anti-nuclear and antiphospholipid antibodies. COVID-19 Reverse transcriptase-polymerase chain reaction (RT-PCR) on Nasopharyngeal swab and serology were also negative	Oxford-AstraZeneca COVID-19 vaccine	1	NA	NA	Dumitru [49]
Fine et al. [50]	Showed marked edema and macular scan revealed the presence of multiple small hyperreflective spots within the inner retinal layers	NA	The basic laboratory workup including complete blood count, erythrocyte sedimentation rate, and C-reactive protein was normal. Contrast-enhanced magnetic resonance imaging (MRI) brain and orbit showed a focal bulge of optic disc on the left side with mild diffusion restriction on diffusion weighted imaging (DWI)	COVAXIN by Bharat Biotech,	1	NA	NA	Fine et al. [50]
Gliem et al. [51]	Several focal disruptions of the ellipsoid and interdigitation zones corresponding to the dots and vertical hyperreflective lines extending from the retinal pigment epithelium, mostly at the fovea	NA	Blue-light fundus autofluorescence images showed hyper- auto fluorescent spots	BNT162b2 mRNA vaccine	2	NA	NA	Gliem et al. [51]
Gross et al. [52]	SRD and choroidal thickening OU	Hypofluorescence in the early phase and hyperfluorescence in the late phase corresponding to the cream-white lesions OU	FAF examination did not reveal any abnormal findings. ICGA revealed sharply marginated hypofluorescent dots of various sizes throughout the mid-venous and late phases OU	Pfizer-BioNTech-BioNTech)	2	NA	NA	Gross et al. [52]
Haseeb et al. [53]	Tomography revealed focal areas of parafoveal hyperreflective bands in the outer retinal in OU without retinal thickening	NA	Fundus autofluorescence showed petaloid faintly hypoautofluorescent lesions in the OD	adenovector corona- virus vaccine	1	NA	NA	Haseeb et al. [53]
Helal et al. [54]	Hyperreflectivity of the outer plexiform layer, Henle fiber layer, and outer nuclear layer. Nasal to the unchanged pigment epithelium detachment	NA	OCT angiography at the level of the deep capillary plexus showed a Semilunar area of flow void corresponding with the hyperreflective deep spectral-domain OCT lesion	Sinopharm	NA	NA	NA	Helal et al. [54]
Khochtali et al. [74]	NA	NA	NA	BNT162b2 mRNA	NA	5	NA	Khochtali et al. [74]
Khochtali et al. [74]	NA	NA	NA	BNT162b2 mRNA	2	30	NA	Khochtali et al. [74]
Kim et al. [55]	EDI‐OCT showed a dome‐shaped serous detachment of the macula with significant SRF and PEDs in the superior macula of the OD	Normal	Fundus autofluorescence revealed the absence of normal central hypoautofluorescence in the OD There was a hypoautofluorescence area superior to the macula	Sinopharm	1	NA	NA	Kim et al. [55]
Li et al. [56]	Demonstrated EZ disruption with intraretinal hemorrhages and hyperreflectivity in the ONL and OPL bilaterally	Demonstrated normal AV transit with no leakage in either eye	NA	Moderna COVID-19 vaccine	2	NA	NA	Li et al. [56]
McMichael et al. [57]	OD showed hyper-reflectivity at the level of outer plexiform and outer nuclear layer	Was not done as the patient did not give consent	DCP showed a well maintained FAZ, but there were areas of hyper-reflectivity on enface imaging at the same level with distortion of FAZ. CC showed hyporeflective areas with loss of coarseness which reduced as the condition improved. FAF and NIRAF were normal Visual fields 24–2 and 10–2 was within normal limits	COVISHIELDTM Vaccination	1	NA	NA	McMichael et al. [57]
Ng et al. [58]	Revealed a dome-shaped macula (DSM) with subretinal fluid and interruption of the ellipsoid zone	Multiple hyperfluorescent dots in a wreath-like pattern,	Late phase indocyanine green angiography revealed hypofluorescent dots in areas corresponding to the autofluorescent fundus lesions. The 30–2 Humphrey visual field test showed enlargement of the blind spot	BNT162b2 vaccine	2	NA	NA	Ng et al. [58]
Papasavvas et al. [75]	Ellipsoid disruption corresponding to the white lesions with involvement under his fovea, likely causing his reduced vision	NA	FAF imaging demonstrated typical appearance for MEWDS in the OD with multiple hyperautofluorescent lesions concentrated and coalescing around the disc	Pfizer-BioNTech-BioNTech (BNT162b2) COVID-19 vaccine	2	NA	NA	Papasavvas et al. [75]
Papasavvas et al. [75]	Focal areas of ellipsoid zone disruption corresponding to the subretinal white lesions on examination, also involving the fovea in her case	Demonstrated both early and late hyperfluorescence of the lesions, subtle wreath-like vascular changes around the fovea and late disc leakage	FAF imaging demonstrates similar hyperautofluorescent lesions concentrated around the disc	Pfizer-BioNTech-BioNTech (BNT162b2) COVID-19	2	NA	NA	Papasavvas et al. [75]
Prieto-Peña et al. [18]	The OS revealed multifocal areas of ellipsoid zone (EZ) loss with associated areas of outer retinal hyperreflectivity	The OS demonstrated hyperfluorescent staining of the corresponding areas	FAF of the OS revealed a confluent area of hyperautofluorescence in the posterior pole with scattered hyperautofluorescent dots. Late phase ICG imaging showed diffuse hypocyanescent spots in the posterior pole	mRNA-1273 COVID-19 vaccine (Moderna)	2	NA	NA	Prieto-Peña et al. [18]
Roy et al. [25]	Revealed thickening of the retina’s outermost layers plus foveal RPE uniformity loss	Presented a wreathlike pattern, matching the lesions at the level of the RPE	The fundus image showed white spots presenting as hyperautofluorescent dots on the fundus autofluorescence (FAF)	COVID-19 Vaccination (Sinovac-CoroNAVac, Sinovac/China NAtioNAl Pharmaceutical Group)	1	NA	NA	Roy et al. [25]
Sasajima et al. [59]	Corresponding parafoveal foci of outer nuclear layer hyperreflectivity with granularity of the underlying ellipsoid	NA	NA	Pfizer-BioNTech-BioNTech COVID-19	2	NA	NA	Sasajima et al. [59]
Shah et al. [60]	Normal foveal contour with hyperreflective lesions involving the nerve fiber layer with back shadowing. There was multiple small hyperreflective spots within the ganglion cell layer and faint outer plexiform layer hyperreflective changes with focal loss of external limiting membrane and intact IS/OS junction in both the eyes	NA	The fundus auto‐fluorescence revealed a normal pattern	SARS‐CoV‐2 vaccination (Covishield)	1	NA	NA	Shah et al. [60]
Sriwijitalai et al. [76]	Some intraretinal hyperreflective lesions above the EZ line. The EZ was relatively preserved than that at the peripapillary region. Besides, there was no hyper autofluorescence change in the central foveal region;	NA	In the peripapillary area and posterior deep retina. These lesions exhibited hyperautofluorescence on the FAF	Oxford - AstraZeneca COVID-19 (AZ)	NA	NA	NA	Sriwijitalai et al. [76]
Sutandi et al. [61]	Optic nerve edema OU	Severe leakage from disc OD with mild leakage from disc OS and wreath-like choroidal hyperfluorescence of posterior pole OU	Funduscopy (OU disc edema, & multifocal well circumscribed deep chorioretinal white lesions in periphery)	Pfizer-BioNTech mRNA 3 weeks ago	1	21	NA	Sutandi et al. [61]
Tomkins-Netzer et al. [62]	Nerve with extension to the midperiphery. OCT demonstrated impaired EZ of the posterior retina. AF demonstrated multifocal hyperautofluorescent spots. FFA of the OS showed early hyperfluorescence with minimal late staining, with patchy staining with no obvious activity	Showed multiple round lesions in the posterior pole with early hyper-fluorescence and late staining, along with several patchy staining at the level of the outer retina and choroid supertemporal to the macule	FFA and AF revealed hyperfluorescence corresponding to the area of the retina in the region of EZ abnormalities	SARS-CoV-2 vaccine (CoronaVac vaccine, Sinovac Life Sciences, China)	1	NA	NA	Tomkins-Netzer et al. [62]
Wang et al. [63]	Punctate hyperreflective lesions of variable sizes in the outer retina and diffuse disruption in EZ at macula	Early punctate hyperfluorescence in a wreath-like pattern with late staining;	FAF there were mixed multiple hypofluorescent spots surrounded by small hyperfluorescent circles and scattered hyperfluorescent lesions, which were concentrated around optic disc and posterior pole in OD, ICGA indicated multiple hypofluorescent spots of various sizes especially in the late phases	BNT162b2 mRNA COVID-19 vaccine (Pfizer-BioNTech-BioNTech)	2	NA	NA	Wang et al. [63]
Yang et al. [64]	Obvious focal area of diffuse hyperreflectivity in the outer plexiform layer and the outer nuclear layer with outer retinal layers showing granular disruption of ellipsoid zone and interdigitation zone	NA	OCT-A: There was no obvious signal attenuation in DCP. Multifocal electroretinograms did not highlight any abnormality as well	Vaccine AstraZeneca	1	NA	Myalgia, and joint pains as her main symptoms. She also had pins and needles like sensation at the injection site and slight difficulty in walking straight	Yang et al. [64]

ADHD: Attention Deficit Hyperactivity Disorder, AF: Autofluorescence, CC: Choriocapillaris, CNV: Choroidal Neovascularization, CP: Color Photography, CT: Computerized tomography DCP: Deep capillary plexus, DM: Diabetic Mellitus, DVT: Deep Vein Thrombosis, ELM: External Limiting Membrane, ERM: Epiretinal Membrane, EZ: Ellipsoid Zone, FP: Fundus Photography, FAF: Fundus Autofluorescence, HCV: Hepatitis C Virus Infection, HFL: Henle's Fiber Layer, HTN: Hypertension, ICG: Indocyanine Green Angiography, ICGA: Indocyanine Green Angiography, IgG: Immunoglobulin G IUD: Intrauterine Device, IR: Infrared Imaging, IRF: Intraretinal Fluid, IZ: Interdigitation Zone, LMWH: Low Molecular Weight Heparin, NPDR: Non-Proliferative Diabetic Retinopathy, OD: Right eye, OPL: Outer Plexiform Layer, ONL: Outer Nuclear Layer, OCP: Oral Contraceptive Pills, OS: Left eye, OU: Both eyes, PCR: Polymerase chain reaction, PPV: Pars Plana Vitrectomy, PR: Photoreceptors, RD: Retinal Detachment, RPE: Retinal Pigment Epithelium, SB: Scleral Buckle, VF: Visual Field

The reported COVID-19 vaccines-associated WDS were MEWDS (n = 33, 51.56%), AMN (n = 22 34.38%), APMPPE (n = 6, 9.38%), paracentral acute middle maculopathy (n = 1, 1.4%), multifocal choroiditis (n = 1, 1.56%), persistent placoid maculopathy (n = 1, 1.56%), and punctate inner choroidopathy (n = 1, 1.56%). Forty-five (70.31%) cases were unilateral while twenty 19 were bilateral (29.69%). Interestingly, all female cases presented with unilateral involvement except one case was bilateral. On the other hand, all males developed bilateral WDS. The mean duration of the WDS presentation was 18.88 ± 41.06 days while the median was 7 days. Twenty-six (40.63%) patients complained of blurred vision, followed by paracentral scotoma (n = 19, 15.63%), and photopsia in (n = 8, 12.50%) and visual field disturbance reported in six patients (n = 6, 9.38%). Regarding the treatment, nineteen patients (29.69%) received steroids. Among them, nine (47.37%) reported improvement from the baseline, four (21.05%) experienced complete resolution, one (5.26%) showed no improvement, and the outcome was not reported for five patients (26.32%). In addition, eleven patients (17.19%) were managed by observation only. Of them, five (45.45%) improved and the other six (54.55%) patients reported complete resolution. The management was not reported in 31 subjects, The average duration of treatment was 23.43 ± 23.46 days. The average duration of follow-up is 9.64 ± 13.43 weeks. (Table 2).

**Table 2:** Clinical description of included articles and related management and outcomes

Author (ID)	ID	Diagnosis	Laterality	Duration of the diagnosed disease	Associated symptoms	Management	Outcomes	Follow-up duration
Alhabshan et al. [35]	13	MEWDS	Unilateral	4 days	Blurred vision, and enlarged blind spot	Observation	Improvement, complication: NA	8 weeks
Book et al. [65]	14	TB Choroiditis Recurrence	Unilateral	21 days	Blurred vision	Intravitreal injection dexamethasone (0.7 mg)	Improvement VA 20/400, complication: NA	2 weeks
Book et al. [65]	14	TB Choroiditis Recurrence	Unilateral	14 days	Floaters	Intravitreal injection dexamethasone (0.7 mg)	Marked resolution of choroiditis lesions while VA remained stable at 20/20., complication: NA	2 weeks
Capuano et al. [20]	15	APMPPE	Unilateral then Bilateral	NA	Blurred vision	Observation	Resolution, BCVA 20/20, complication: NA	5 weeks
Conrady et al. ([26]	19	MEWDS	Unilateral	7 days	Blurred vision, scotoma, and photopsia	Observation	Spontaneous resolution, VA 20/20, complication: NA	68 weeks
David et al. [36]	21	APMPPE	Unilateral	3 days	Blurred vision, and Temporal VF defect OS	OS Retrobulbar Triamcinolone 20 mg injection & OU Prednisolone acetate 1% drops 4 times daily	FA: early hypofluorescence corresponding to placoid lesions followed by late irregular hyperfluorescent staining), complication: none	1 week
De Salvo et al. [37]	22	AMN	Bilateral	30 days	Blurred vision	Oral prednisone started at 60 mg daily and tapered over 3 weeks	Mild improvement, OD 20/80 OS 20/100, complication: central scotoma persistence	8 weeks
El Matri et al. [38]	25	AMN	Unilateral	NA	Paracentral scotoma	NA	NA, complication: NA	NA
Fekri et al. [66]	26	MEWDS	Unilateral	NA	Blurred vision and visual field disturbance	Observation	20/20, complete resolution, complication: NA	NA
Fekri et al. [66]	26	MEWDS	Unilateral	NA	Blurred vision and visual field disturbance	Observation	20/20, complete resolution, complication: NA	NA
Fekri et al. [66]	26	MEWDS	Unilateral	NA	Blurred vision	Observation	20/20, complete resolution, complication: NA	NA
Fekri et al. [66]	26	AMN	Bilateral	NA	Visual field disturbance	Observation	20/20 OU, significant improvement, complication: NA	NA
Fischer et al. [39]	27	AMN	Bilateral	NA	Paracentral scotoma	NA	NA, complication: NA	NA
Gabrielle et al. [17]	29	MEWDS	Unilateral	NA	Blurred vision, and photopsia	NA	20/25, FAF significant regression, OCT normal, complication: NA	4 weeks
Gabrielle et al. [17]	29	MEWDS	Unilateral	7 days	Photopsia	NA	20/20, no lesions, complication: NA	12 weeks
Gabrielle et al. [17]	29	MEWDS	Unilateral	NA	Enlarged blind spot, and photopsia	NA	Resolution, complication: NA	16 weeks
Hawley et al. [40]	36	Persistent Placoid Maculopathy	Bilateral	NA	Blurred vision	High dose oral prednisolone of a slow tapering regime then Mycophenolate mofetil long term	3/60 OD CF OS, complication: NA	NA
Patel et al. [41]	62	AMN	Bilateral	NA	Paracentral scotoma	40 mg prednisolone daily for one week followed by a dose of 20 mg for another week	OCT Regression, microperimetry scotoma improvement, complication: NA	15 weeks
Rennie et al. [42]	71	AMN	Bilateral	14 days	Scotomas	Oral prednisolone 25 mg/day	VF OU improvement, resolved symptoms, OCT partial improvement, complication: NA	2 weeks
Vinzamuri et al. [67]	79	AMN	Unilateral	NA	Fortifications	Observation	VA 20/16, visual symptoms same, SD-OCT revealed the resolution of the hyperreflectivity in the OPL and ONL but unchanged disruption of the photoreceptor layers, complication: NA	8 weeks
Vinzamuri et al. [67]	79	AMN	Unilateral	NA	Fortifications, and paracentral scotoma	Observation	VA 20/20, visual symptoms same, SD-OCT revealed the resolution of the hyperreflectivity in the OPL and ONL but unchanged disruption of the photoreceptor layers, complication: NA	15 weeks
Virgo et al. [43]	80	AMN	Bilateral	NA	Paracentral scotoma, and black spots	NA	NA, complication: NA	NA
Xu et al. ([68]	83	MEWDS	Unilateral	3 days	Blurred vision	NA	Spontaneous resolution, complication: NA	6 weeks
Xu et al. [68]	83	MEWDS	Unilateral	6 days	Blurred vision	NA	Spontaneous resolution, complication: NA	6 weeks
Zaheer et al. [69]	87	AMN	Bilateral	NA	Scotoma	NA	NA, complication: NA	NA
Fowler et al. [44]	91	Multifocal Choroiditis	Bilateral	1 day	Blurred vision, and OS nasal redness, OD floater	Oral prednisolone 100 mg daily (1 mg per kilogram of body weight) tapering by 10 mg every week	Significant improvement. VA 6/6, N6 in OU. Fundus examination showed the choroiditis had resolved significantly, complication: NA	1.6 weeks
**Mélanie H. et al. **[45]	104	Panuveitis with Occlusive Vasculitis	Bilateral	7 days	Blurred vision, floaters, photophobia, burning sensation	Prednisolone 1% every hour OU with a taper with cyclopentolate twice daily and dexamethasone ointment at bedtime OU for five days THEN timolol daily OU was added THEN 8 days later prednisolone 1% dosing was increased every hour with cyclopentolate 1% and dexamethasone ointment at bedtime THEN Oral prednisone was started at 50 mg daily with a taper in addition to the topical drops. prednisolone 1% every hour. At follow-up 4.5 months later, the patient was successfully tapered to 5 mg of oral prednisone and prednisolone 1% twice daily with timolol once daily for IOP control	PHVA was 20/30 + 1 OD and 20/30 + 2 OS, complication: fluorescein angiography showed a peripheral avascular zone with staining temporally OU, the same asymptomatic peripapillary pigment epithelial detachment OD, and suspicion of a single peripheral neovascularization OD so follow up with vitreoretinal specialist	18 weeks
Ahmed et al. [46]	110	MEWDS	Unilateral	3 days	Blurred vision	Neomycin & Betamethasone sodium phosphate	VA same, fundus lesions absent, complication: NA	8 weeks
Ahmed et al. [70]	111	AMN	Unilateral	3 days	Visual field disturbance	NA	NA, complication: NA	NA
Azar et al. [12]	113	APMPPE	Unilateral	NA	Sudden blind spots	Observation for 42 days till anterior vitritis developed then oral prednisolone tapered for 5 weeks	20/20 OU, placoid lesions were replaced by pigmented scars, and the anterior vitritis was completely resolved, complication: NA	16 weeks
Azar et al. [88]	114	AMN	Unilateral	NA	Temporal paracentral scotoma	NA	NA, complication: NA	NA
Azar et al. [88]	114	AMN	Unilateral	NA	Blurred vision, and photopsia	NA	Resolution, complication: NA	8 weeks
Cuadros Sánchezet al [47]	127	Neuroretinitis	Unilateral	30 days	Blurred vision (inferior field blurry)	1 g of intravenous methylprednisolone daily for 3 days, followed by oral prednisolone with a tapering dosage	CF OD 20/20 OS, optic nerve swelling in OD resolved promptly after this treatment., complication: the amount of subretinal fluid slightly increased. Follow-up fundus photography showed mild pallor without swelling in the right optic nerve head	48 weeks
Cunha Ângelo et al. [71]	128	MEWDS	Unilateral	NA	NA	Oral steroids	NA, complication: NA	NA
Cunningham et al. [72]	129	MEWDS	Unilateral	17 days	NA	NA	NA, complication: NA	NA
Cunningham et al. [72]	129	MEWDS	Unilateral	30 days	NA	NA	NA, complication: NA	NA
Cunningham et al. [72]	129	MEWDS	Unilateral	3 days	NA	NA	NA, complication: NA	NA
Cunningham et al. [72]	129	MEWDS	Bilateral	10 days	NA	NA	NA, complication: NA	NA
Cunningham et al. [72]	129	MEWDS	Unilateral	14 days	NA	NA	NA, complication: NA	NA
Cunningham et al. [72]	129	MEWDS	Unilateral	14 days	NA	NA	NA, complication: NA	NA
Cunningham et al. [72]	129	MEWDS	Unilateral	7 days	NA	NA	NA, complication: NA	NA
Cunningham et al. [72]	129	MEWDS	Unilateral	14 days	NA	NA	NA, complication: NA	NA
Cunningham et al. [72]	129	PIC	Unilateral	210 days	NA	NA	NA, complication: NA	NA
Cunningham et al. [72]	129	APMPPE	Bilateral	7 days	NA	NA	NA, complication: NA	NA
Da Silva et al. [24]	131	MEWDS	Unilateral	NA	NA	Observation	20/20, complete resolution, complication: NA	4 weeks
Detrick et al. [73]	137	AMN	Unilateral	NA	Sudden onset scotoma	NA	NA, complication: NA	NA
Diafas Et al [48]	139	APMPPE	Bilateral	14 days	Blurred vision	Intravenous methylprednisolone therapy for 3 days, followed by oral prednisolone	NA, complication: NA	NA
Dumitru [49]	142	AMN	Unilateral	42 days	Sudden onset of four central scotomas OS	NA	Metrovision visual field 10–2 testing showed improvement. The SD-OCT showed thickening of ONL and thinning of interdigitation zone without focal interruption of EZ/IZ, complication: only three scotomas remaining	6 weeks
Fine et al. [50]	151	Neuroretinitis	Unilateral	NA	Sudden onset of painful diminution of vision OS	Pulse steroid therapy (IV Methylprednisolone 1 g for	NA, complication: NA	6 weeks
Gliem et al. [51]	156	MEWDS	Unilateral	NA	Blurred vision, and central scotoma	NA	Multimodal imaging showed resolution of the retinal lesions on fundoscopy image and optical, complication: NA	8 weeks
Gross et al. [52]	160	APMPPE	Bilateral	4 days	Blurred vision	Prednisolone (PSL; 100 mg for 3 days following 80 mg for 2 days). steroid pulse therapy (1,000 mg of methylprednisolone daily for 3 days) was performed, followed by 60 mg of oral PSL	No recurrences, and no retinal scar lesions, complication: NA	48 weeks
Haseeb et al. [53]	166	AMN	Bilateral	NA	Paracentral scotomas OU	Conservative management	NA, complication: NA	NA
Helal et al. [54]	169	AMN	Unilateral	NA	NA	NA	NA, complication: NA	8 weeks
Khochtali et al. [74]	175	MEWDS	Unilateral	NA	Blurred vision, visual field disturbance, and photopsia	Observation	20/20 Significant improvement, complication: NA	NA
Khochtali et al. [74]	175	MEWDS	Unilateral	NA	Blurred vision, visual field disturbance, and photopsia	Observation	20/20 Significant improvement, complication: NA	NA
Kim et al. [55]	177	Acute Central Serous Chorioretinopathy	Unilateral	NA	Blurred vision	NA	NA, complication: NA	NA
Li et al. [56]	182	AMN	Bilateral	NA	Paracentral scotomas	options for a trial of a short course of steroids were discussed, and the treatment was initiated with Difluorinated 0.05% four times a day OU and a single 20 mg dose of oral prednisone. Options for oral steroids were discussed with the patient and family, but it was decided not to pursue any longer duration of oral prednisone	NA, complication: NA	NA
McMichael et al. [57]	187	AMN	Unilateral	NA	Shadow and a week later there was a new scotoma in OD	NA	NA, complication: NA	NA
Ng et al. [58]	196	MEWDS	Unilateral	NA	Enlarging dark spot over her right central vision	15 mg of oral prednisone per day, which was tapered over 2 weeks. Additionally, she was administered intravitreal bevacizumab	Symptoms improved with increased visual acuity to 20 / 50. The fundus lesions resolved further, complication: subfoveal hyperreflective material was still observed on OCT	1.4 weeks
Papasavvas et al. [75]	202	MEWDS	Unilateral	NA	Blurred vision, and black floaters and lights in OD	Oral prednisolone	Largely resolved, complication: NA	2 weeks
Papasavvas et al. [75]	202	MEWDS	Unilateral	NA	Blurred vision	With 40 mg of oral prednisolone	Completely resolved with resolution of her symptoms and restoration of BCVA of 20/20 in OU, complication: NA	2 weeks
Prieto-Peña et al. [18]	203	MEWDS	Unilateral	NA	Temporal scotoma	60 mg of oral prednisone	NA, complication: NA	NA
Roy et al. [25]	210	MEWDS	Unilateral	NA	Photopsia	80-mg/day oral prednisone	Improvement in visual acuity (20/20 OU), complication: NA	4 weeks
Sasajima et al. [59]	213	AMN	Bilateral	NA	Paracentral scotomas OU	Treated empirically with 60mg oral prednisone and 50mg diphenhydramine	NA, complication: NA	NA
Shah et al. [60]	217	PAMM and AMN	Bilateral	NA	Blurred vision, and black spots in vision, and reduced brightness of vision OU	NA	Slight improvement of brightness sensitivity, vision was 6/6 OU, complication: black spots in central field of Vision	3 weeks
Sriwijitalai et al. [76]	220	MEWDS	Unilateral	NA	Central scotoma OD	A low‐dose oral steroid of 10mg prednisolone per day	BCVA recovered to 20/20, complication: NA	7 weeks
Sutandi et al. [61]	223	MEWDS	Bilateral	NA	Blurred vision	Valacyclovir 100 mg twice daily for 10 days, Acetazolamide 750 mg twice daily for 30 days	VA 20/20, resolved edema and vitreous hemorrhage, complication: NA	4 weeks
Tomkins-Netzer et al. [62]	225	MEWDS	Unilateral	NA	Blurred vision	Antiviral drug (acyclovir) combined with Vitamin B2 and Vitamin C	BCVA had improved to 20/20 OS, complication” NA	8 weeks
Wang et al. [63]	230	MEWDS	Unilateral	5 days	Blurred vision, central visual field loss; central scotoma and paracentral islands of sensitivity loss in OD	No treatment	Multifocal white dots had disappeared in OD and were almost invisible on fundus photography, her BCVA was 0.4 (20/50) OD and 1.2 (20/16) OS at that time, complication: RPE color changes at posterior pole along with active vitritis were seen, multifocal ERGs showed decreased retinal response with low-amplitude density over the entire field in OD and normal retinal response amplitude density in OS	1 week
Yang et al. [64]	234	AMN	Unilateral	NA	2 paracentral scotomas OD	NA	Vision was stable at 6/5 OU, complication: no change in the size or number of her scotomas	4 weeks

APMPPE: Acute posterior multifocal placoid pigment epitheliopathy, BCVA: Best-Corrected Visual Acuity, CF: Counting fingers, ERG: Electroretinogram, FA: Fluorescein Angiography, MEWDS: Multiple Evanescent White Dot Syndrome, OCT: Optical Coherence Tomography, OD: Right eye, ONL: Outer Nuclear Layer, OPL: Outer Plexiform Layer, OS: Left eye, OU: Both eyes, PHVA: Pinhole Visual Acuity, RPE: Retinal pigment epithelium, SD-OCT: Spectral-Domain Optical Coherence Tomography, TB: Tuberculosis, VA: Visual Acuity, and VF: Visual Field

Regarding the patients’ previous ophthalmological history, six (9.38%) patients reported a previous history of ophthalmological issues. Myopia was present in five patients (83.33%), previous ocular surgeries/treatments reported in three patients (50%), and specific eye conditions (central serous chorioretinopathy with chronic serous pigment epithelial detachment and a previous episode of MEWDS) were found in two patients (33.33%). The mean baseline visual acuity (LogMAR) was 1.02 ± 0.43. (Table 1).

**Fig. 2:**
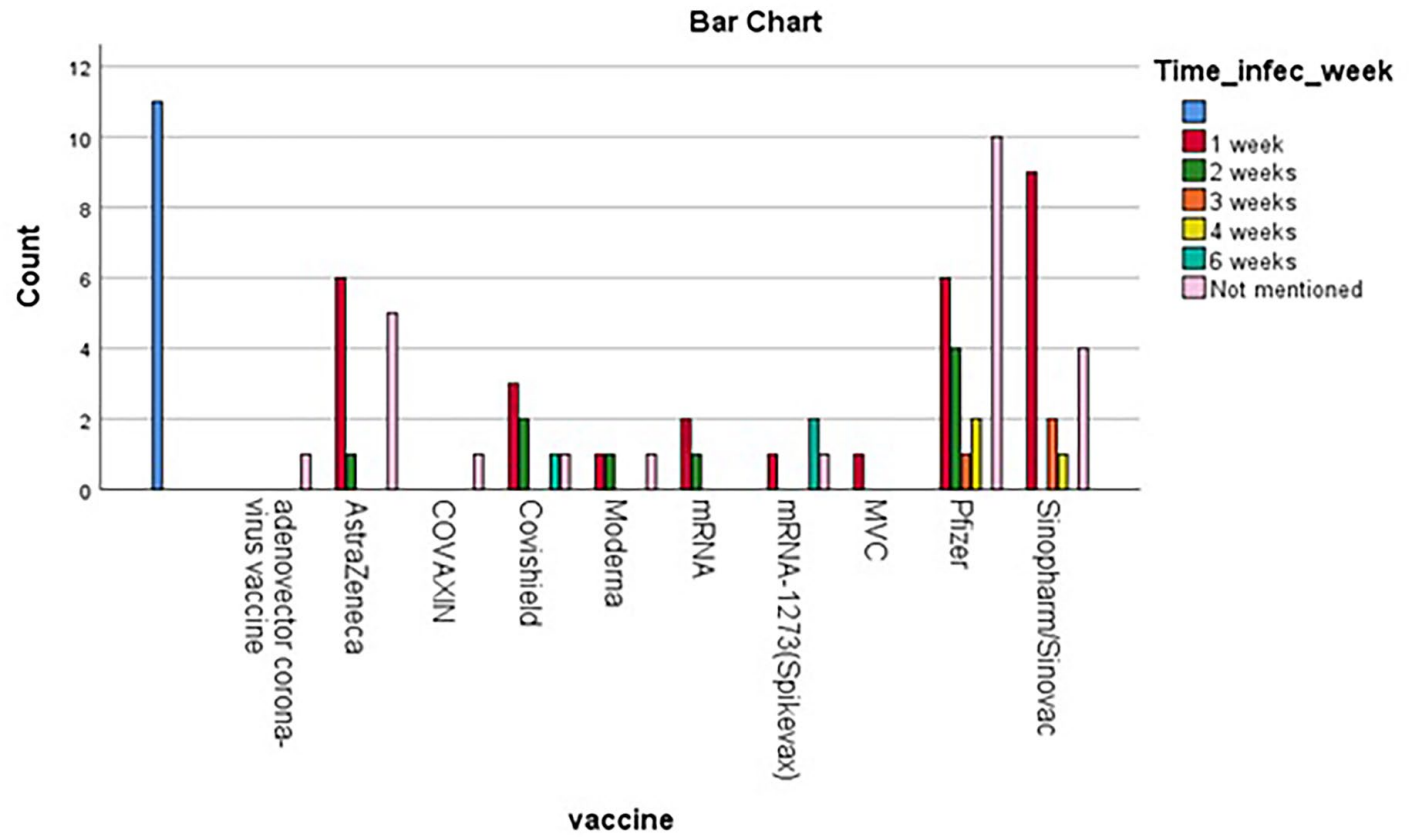
shows the mean duration from administration the vaccine till the onset of WDS symptoms

Optical coherence tomography (OCT) was conducted in forty-six fifty patients (71.88%). One patient only had normal OCT (2.17%) while the others reported mainly EZ disruption (n = 27, 58.70%), lesions and hyperreflective spots (n = 23, 50.00%), outer retinal layer abnormalities (n = 19, 41.30%), and subretinal fluid and detachments (n = 7, 15.22%). Moreover, fluorescein angiography (FA) was performed in twenty-three patients (35.94%) which were normal in three patients (13.04%) while the other patients reported hyperfluorescence with wreath-like pattern (n = 10, 43.48%), early hypofluorescence and late hyperfluorescence (n = 10, 43.48%), and late staining or leakage (n = 9, 39.13%). Fundus autofluorescence (FAF) was reported in thirteen patients (20.31%) which was normal in one patient (7.69%), and the other patients reported hyper autofluorescence (n = 9, 69.23%), while hypoautofluorescent (n = 10, 76.92%%). The mean number of doses that were administered to the patients was 1.47 ± 0.54. The mean duration from taking the vaccine till the onset of symptoms was 9.60 ± 10.66 days (mean ± SD). (Fig. 2) Complications of vaccination were reported in twelve patients (18.75%) including, but not limited to, flu-like symptoms (n = 4, 33.33%), and pain at the injection site (n = 4, 33.33%). (Table 1) Long-term ocular complications were observed in 13.4% of patients (n = 11). Most of them were persistent scotoma (n = 4, 36.7%). (Supplementary Table 3).

## Discussion

Since the emergence of COVID-19 vaccines, many adverse events have been recognized globally. Of these adverse events, different types of WDS had been reported in the literature. Our results reviewed 82 cases received different COVID-19 vaccines. The most reported vaccine used was Pfizer (n = 23, 28%) in which showed a predominance of MEWDS (n = 15, 65.2%) and the rest of cases (n = 4, 17%) were diagnosed as AMPPE. AstraZeneca was seen in a total of 12 cases (14.6%), 10 cases of them diagnosed with AMN. Finally, Sinovac was administered in 16 cases (19%) of which 10 of them were associated with MEWDS and three of them were associated with AMN. Therefore, our data showed that Pfizer and AstraZeneca vaccine are associated mainly with MEWDS, and AMN, respectively. This could be explained by the fact that MEWDS is believed to be of an autoimmune nature given its autoimmune associations [11]. Pfizer–BioNTech vaccine produces an additional CD8 T-cell immune response triggering autoimmune reactions. [77] Nevertheless, this finding could also be explained by the dominance of the Pfizer-BioNTech vaccine over other COVID-19 vaccine types in the number of given doses worldwide [78]. Furthermore, AMN is hypothesized to be a systemic autoimmune disease that causes small-vessel occlusion due to micro-thrombi production, leading to ischemic retinopathy [79]. It has been reported that AstraZeneca vaccine provides protection against SARS-CoV-2 infection through immune-mediated mechanisms which are believed to cause thrombosis through an activation of platelets, immune cells, and hypercoagulability factors [10].

Our review included subjects with a mean age of 32.79 ± 14.81 years which is the typical age of WDS reported in the literature [82]. Because of the autoimmunity nature of WDS, it tends to occur more frequently in females [83]. Although it's unclear what's causing this trend, there is growing evidence that sex hormones affect the immune response, with estrogen enhancing and androgens suppressing it [84]. Moreover, it has been hypothesized that estrogen is crucial for the development and function of Th17 cells in addition to IL-17 generation [85].Our results coincide with this trend, showing that COVID-19 vaccine-associated WDS were more likely to occur in females than in males (77.5% vs. 21.1%).

The mean of the duration from taking the vaccine till the onset of symptoms was 10.06 ± 11.37 days (mean ± SD). In the literature, ocular adverse effects of COVID-19 vaccines, in general, happened in the first 10 days after administration of vaccines [86] .This temporal association could be explained by the fact that vaccine-related antibodies, that promote immune response including hypercoagulability, appear maximally within the first 5–10 days after vaccination, and disappear within 100 days [87]. In addition, 58.3% of WDS after AstraZeneca vaccine administration occurred within the first week, and all of them were AMN. Pfizer–BioNTech vaccine cases also were seen most frequently in the first week (46.1%) and second week (30.8%). On the other hand, Covishield and mRNA Spikvax-associated WDS were observed within 2 months of administration. (Supplementary Table 4).

Regarding OCT, VF and FAF findings in our study, the results were consistent with previously reported literature. The SD-OCT appearance of MEWDS is that of disruption mainly of the ellipsoid zone and interdigitation zone complex in the fovea and it is sometimes associated with reflective focal lesions that crossed the external limiting membrane line [80] and FA reveals early punctate hyperfluorescence in a wreath-like pattern and late staining, in areas corresponding to the white dots. This hyperfluorescence may be due to dilated retinal microcirculation in the middle or deep retinal capillary plexus [81].

Regarding VF complications, a total of nine patients (9.7%) reported complications of VF ch were mainly associated with AMN (n = 5, 62.5%) in the form of the typical VF defects (paracentral and temporal scotoma). Other 2 cases (n = 2, 25%) were associated with MEWDS in the form of enlarged blind spot and paracentral scotoma. Lastly, one case (n = 1, 12.5%) was associated with neuroretinitis in the form of superior scotoma and generalized field defect. Our results showed further analysis of the types of vaccine causing VF defect. Four cases of paracentral scotoma were found. Two of them were associated with AstraZeneca vaccine, one case was associated with MVC COVID-19 and Sinopharm vaccines, respectively. Enlarged blind spots and inferotemporal scotomas were seen in only one case, respectively, due to Spikevax vaccine. Interestingly, Pfizer–BioNTech vaccine has not been reported to cause VF defects.

Overall, the etiology of WDS is still uncertain and the emergence of cases under COVID-19 vaccines may shed some light on the exact pathogenesis of these syndromes. According to the WHO, as of the 21st of September 2023, 13.5 billion COVID-19 vaccine doses have been administered globally, and 27,338 are now administered each day [1]. Therefore, this possible association between COVID-19 vaccines and WDS might be a coincidence. In addition, the nature of case reports and series may introduce bias and limit the generalizability of our findings raising questionable associations. Further research is recommended to investigate these possible associations. The large number of studies included increases the discrepancy in reporting between different studies, hence a large group study could mitigate this effect and unify the reporting criteria for these syndromes.

## Conclusion

Our review summarizes the occurrence of COVID-19 vaccination-associated WDS, which is more likely to occur among middle-aged females. Our findings indicate a possible association between COVID-19 vaccines and WDS, but this association is limited by the quality and number of available studies. The clinicians should be aware enough of this possible association and report them immediately upon the identification of similar cases for better implementation of the evidence. Further studies are needed for better determination of the incidence, risk factors, characteristics, and management of these syndromes.

## Supplementary Information

Below is the link to the electronic supplementary material.

Supplementary file1 (DOCX 279 KB)

## Data Availability

The datasets used and/or analysed during the current study are available from the corresponding author on reasonable request.
