# Supplementary material for: The characteristics of white dot syndromes following COVID-19 Vaccines: a systematic review
Source: Int Ophthalmol. 2024 Apr 23;44(1):189. doi: 10.1007/s10792-024-03119-4 (PMC11039548; doi:10.1007/s10792-024-03119-4)
Supplement: Supplementary file 1 — Supplementary file1 (DOCX 279 KB) [file 10792_2024_3119_MOESM1_ESM.docx]

**Supplementary Table-1.** The quality assessment and overall appraisal of included case reports.

| ID | Country | Study design | Were patient’s demographic characteristics clearly described?(Yes,No, Unclear, Not applicable) | Was the patient’s history clearly described and presented as a timeline?(Yes,No, Unclear, Not applicable) | Was the current clinical condition of the patient on presentation clearly described?(Yes,No, Unclear, Not applicable) | Were diagnostic tests or assessment methods and the results clearly described?(Yes,No, Unclear, Not applicable) | Was the intervention(s) or treatment procedure(s) clearly described?(Yes,No, Unclear, Not applicable) | Was the post-intervention clinical condition clearly described?(Yes,No, Unclear, Not applicable) | Were adverse events (harms) or unanticipated events identified and described?(Yes,No, Unclear, Not applicable) | Does the case report provide takeaway lessons?(Yes,No, Unclear, Not applicable) | Overall appraisal: (Include, Exclude, Seek further info) |
| --- | --- | --- | --- | --- | --- | --- | --- | --- | --- | --- | --- |
| [1] | USA | Case Report | Yes | Yes | Yes | Yes | Not applicable | Not applicable | Not applicable | Yes | Include |
| [2] | Turkey | Case Report | Yes | Yes | Yes | Yes | Not applicable | Not applicable | Not applicable | Yes | Include |
| [3] | India | Case Report | Yes | Yes | Yes | Yes | Not applicable | Not applicable | Not applicable | Yes | Include |
| [4] | USA | Case Report | Yes | Yes | Yes | Yes | Yes | Yes | No | Yes | Include |
| [5] | USA | Case Report | Yes | Yes | Yes | Yes | Yes | Yes | No | Yes | Include |
| [6] | Norway | Case Report | Yes | Yes | Yes | Yes | Yes | Yes | No | No | Include |
| [7] | Germany | Case Report | Yes | Yes | Yes | Yes | Yes | Yes | No | unclear | Include |
| [8] | UK | Case Report | Yes | Yes | Yes | Yes | Yes | Yes | No | Yes | Include |
| [9] | Germany | Case Report | Yes | Yes | Yes | Yes | Yes | Yes | No | Yes | Include |
| [10] | Iran | Case Report | Yes | Yes | Yes | Yes | Yes | Yes | No | Yes | Include |
| [11] | France | Case Report | Yes | Yes | Yes | Yes | Yes | Yes | No | unclear | Include |
| [12] | India | Case Report | Yes | Yes | Yes | Yes | Yes | Yes | No | Yes | Include |
| [13] | CaNAda | Case Report | Yes | Yes | Yes | Yes | Yes | Yes | No | Yes | Include |
| [14] | Japan | Case Report | Yes | No | Yes | Yes | Yes | Yes | No | Yes | Include |
| [15] | USA | Case Report | Yes | Yes | Yes | Yes | Yes | Yes | No | Yes | Include |
| [16] | South Korea | Case Report | Yes | Yes | Yes | Yes | Yes | Yes | No | Yes | Include |
| [17] | Taiwan | Case Report | Yes | Yes | Yes | Yes | Not applicable | Not applicable | Not applicable | Yes | Include |
| [18] | Ireland | Case Report | Yes | Yes | Yes | Yes | Yes | Yes | No | Yes | Include |
| [19] | France | Case Report | Yes | Yes | Yes | Yes | No | Not applicable | Not applicable | Yes | Include |
| [20] | India | Case Report | Yes | No | Yes | Yes | Yes | Yes | No | Yes | Include |
| [21] | France | Case Report | Yes | Yes | Yes | Yes | No | Not applicable | Not applicable | Yes | Include |
| [22] | Japan | Case Report | Yes | No | Yes | Yes | Yes | Yes | No | Yes | Include |
| [23] | USA | Case Report | Yes | Yes | Yes | Yes | No | Not applicable | Not applicable | Yes | Include |
| [24] | UAE | Case Report | Yes | Yes | Yes | Yes | Yes | Yes | No | Yes | Include |
| [25] | Canada | Case Report | Yes | Yes | Yes | Yes | Not applicable | Not applicable | Not applicable | Yes | Include |
| [26] | USA | Case Report | Yes | No | Yes | Yes | Yes | Yes | No | Yes | Include |
| [27] | India | Case Report | Yes | Yes | Yes | Yes | Not applicable | Not applicable | Not applicable | Yes | Include |
| [28] | Seoul, Korea | Case Report | Yes | No | Yes | Yes | Yes | Yes | No | Unclear | Include |
| [29] | USA | Case Report | Yes | Yes | Yes | Yes | Yes | Yes | No | Yes | Include |
| [30] | Brazil. | Case Report | Yes | Yes | Yes | Yes | Yes | Yes | No | Yes | Include |
| [31] | USA | Case Report | Yes | Yes | Yes | Yes | No | Not applicable | Not applicable | Yes | Include |
| [32] | India | Case Report | Yes | No | Yes | Yes | No | Not applicable | Not applicable | Yes | Include |
| [33] | USA | Case Report | Yes | Yes | Yes | Yes | Yes | Yes | No | Yes | Include |
| [34] | China | Case Report | Yes | Yes | Yes | Yes | Yes | Yes | No | Yes | Include |
| [35] | Japan | Case Report | Yes | Yes | Yes | Yes | No | Not applicable | Not applicable | Yes | Include |
| [36] | UK | Case Report | Yes | Yes | Yes | Yes | No | Not applicable | Not applicable | Yes | Include |

**Supplementary Table-2.** The quality assessment and overall appraisal of the included case series.

| ID | Country | Study design | Was the hypothesis/aim/objective of the study clearly stated? (Yes,Partial,No) | Was the study conducted prospectively? (Yes,unclear,No) | Were the cases collected in more than one centre? (Yes,unclear,No) | Were patients recruited consecutively? (Yes,unclear,No) | Were the characteristics of the patients included in the study described? (Yes,Partial,No) | Were the eligibility criteria (i.e. inclusion and exclusion criteria) for entry into the study clearly stated? (Yes,Partial,No) | Did patients enter the study at a similar point in the disease?(Yes,unclear,No) | Was the intervention of interest clearly described? (Yes,Partial,No) | Were additional interventions (co-interventions) clearly described? (Yes,Partial,No) | Were relevant outcome measures established a priori? (Yes,Partial,No) | Were outcome assessors blinded to the intervention that patients received? (Yes,unclear,No) | Were the relevant outcomes measured using appropriate objective/subjective methods? (Yes,Partial,No) | Were the relevant outcome measures made before and after the intervention? (Yes,unclear,No) | Were the statistical tests used to assess the relevant outcomes appropriate? (Yes,unclear,No) | Was follow-up long enough for important events and outcomes to occur? (Yes,unclear,No) | Were losses to follow-up reported? (Yes,unclear,No) | Did the study provided estimates of random variability in the data analysis of relevant outcomes? (Yes,Partial,No) | Were the adverse events reported? (Yes,Partial,No) | Were the conclusions of the study supported by results? (Yes,unclear,No) | Were both competing interests and sources of support for the study reported? (Yes,Partial,No) |
| --- | --- | --- | --- | --- | --- | --- | --- | --- | --- | --- | --- | --- | --- | --- | --- | --- | --- | --- | --- | --- | --- | --- |
| [37] | India | Case Series | Yes | No | No | Unclear | Yes | No | No | Yes | No | No | Unclear | Yes | Yes | Unclear | Unclear | No | No | No | No | Yes |
| [38] | Italy | Case Series | Yes | No | No | Unclear | YEs | Yes | Unclear | Yes | No | No | Unclear | Yes | Yes | Unclear | Unclear | Unclear | No | No | Yes | Yes |
| [39] | Canada | Case Series | Yes | No | Unclear | Unclear | Yes | Yes | No | No | No | Nio | Unclear | Yes | No | Unclear | Unclear | No | No | No | No | Yes |
| [40] | Austria | Case Series | Yes | Unclear | Unclear | Unclear | Yes | No | No | No | No | No | Unclear | Yes | No | Unclear | Unclear | No | No | No | No | Yes |
| [41] | France | Case Series | Yes | Unclear | No | Unclear | Yes | No | No | No | No | No | Unclear | Yes | No | Unclear | Unclear | No | No | No | Yes | Yes |
| [42] | Switzerland | Case Series | Yes | No | Yes | Unclear | Yes | Yes | Unclear | No | No | No | Unclear | Yes | No | Unclear | Unclear | Unclear | No | No | Yes | Yes |
| [43] | Japan | Case Series | Yes | No | Yes | Unclear | Yes | Yes | Unclear | No | No | No | Unclear | Yes | No | Unclear | Unclear | Unclear | No | No | Yes | Yes |
| [44] | The Netherlands | Case Series | Yes | Unclear | No | Unclear | Yes | No | No | No | No | No | Unclear | Yes | No | Unclear | Unclear | No | No | No | Yes | Yes |
| [45] | China | Case Series | Yes | No | No | Yes | Yes | Yes | Unclear | Yes | No | No | Unclear | Yes | Yes | Unclear | Unclear | Unclear | No | No | Yes | Yes |
| [46] | China | Case Series | Yes | No | No | Unclear | Yes | Yes | Unclear | No | No | No | Unclear | No | No | Unclear | Unclear | Unclear | No | No | No | Yes |
| [48] | Israel | Case Series | Yes | No | Yes | Yes | Yes | Yes | No | Yes | No | No | Unclear | Yes | Yes | Unclear | Unclear | No | No | No | Yes | Yes |
| [49] | Australia | Case Series | Yes | Unclear | No | Unclear | Yes | No | No | Yes | No | No | Unclear | Yes | Yes | Unclear | Unclear | No | No | No | No | Yes |

**Supplementary Table-3.** The characteristics of complications depend on the type of vaccine.

| ID | Vaccine | Laterality | Age | Gender | Country | Diagnosis |  | Management | Complications |
| --- | --- | --- | --- | --- | --- | --- | --- | --- | --- |
| [5] | Moderna | Bilateral | 64 | Female | USA | AMN | 30 days | Oral prednisone started at 60 mg daily and tapered over 3 weeks | central scotoma persistence |
| [16] | Pfizer | Unilateral | 83 | Female | South Korea | Neuroretinitis | 30 days | 1 gram of intravenous methylprednisolone daily for 3 days, followed by oral prednisolone with a tapering dosage | the amount of subretinal fluid slightly increased. Follow-up fundus photography showed mild pallor without swelling in the right optic nerve head. generalized field defect in the OD and superior arcuate scotoma in the OS |
| [19] | AstraZeneca | Unilateral | 21 | Female | France | AMN | 42 days | NA | only three scotomas remaining |
| [28] | Pfizer | Unilateral | 33 | Female | South Korea | MEWDS | NA | 15 mg of oral prednisone per day, which was tapered over 2 weeks. Additionally, she was administered intravitreal bevacizumab | subfoveal hyperreflective material was still observed on OCT |
| [35] | Pfizer | Unilateral | 67 | Female | Japan | MEWDS | 5 days | No treatment | RPE color changes at posterior pole along with active vitritis were seen, multifocal ERGs showed decreased retinal response with low-amplitude density over the entire field in OD and normal retinal response amplitude density in OS |
| [36] | AstraZeneca | Unilateral | 22 | Female | UK | AMN | NA | NA | no change in the size or number of her scotomas |
| [32] | Covishield | Bilateral | 34 | Male | India | AMN | NA | NA | black spots in central field of vision |
| [13] | Pfizer | Bilateral | 41 | Male | Canada | Panuveitis with Occlusive Vasculitis | 7 days | Prednisolone 1% every hour OU with a taper | peripheral avascular zone with staining temporally OU suspicion of a single peripheral neovascularization OD |

**Supplementary Table-4.** The onset of white dot syndromes following 2 weeks of vaccine administration.

| ID | Vaccine | The onset of symptoms after vaccine administration | Diagnosis |
| --- | --- | --- | --- |
| [6] | AstraZeneca | 1 week | AMN |
| [7] | AstraZeneca | 1 week | AMN |
| [40] | AstraZeneca | 1 week | AMN |
| [40] | AstraZeneca | 1 week | AMN |
| [11] | AstraZeneca | 1 week | AMN |
| [42] | AstraZeneca | 1 week | AMN |
| [8] | AstraZeneca | 2 weeks | Persistent Placoid Maculopathy |
| [38] | Pfizer | 1 week | MEWDS |
| [38] | Pfizer | 1 week | MEWDS |
| [39] | Pfizer | 1 week | MEWDS |
| [13] | Pfizer | 1 week | Panuveitis with Occlusive Vasculitis |
| [14] | Pfizer | 1 week | MEWDS |
| [16] | Pfizer | 1 week | Neuroretinitis |
| [4] | Pfizer | 2 weeks | APMPPE |
| [43] | Pfizer | 2 weeks | AMN |
| [45] | Pfizer | 2 weeks | MEWDS |
| [15] | Pfizer | 2 weeks | APMPPE |

**References:**

[1] R. Alhabshan and D. Scales, “Multiple Evanescent White Dot Syndrome Developing Three Days following Administration of mRNA-1273 Booster Vaccine: Case Report,” *Case Rep Ophthalmol*, vol. 13, no. 2, 2022, doi: 10.1159/000525687.

[2] F. Atas, M. Kaya, and A. O. Saatci, “Acute Multifocal Placoid Pigment Epitheliopathy-like Presentation following the First Dose of BNT162B2 COVID-19 Vaccination,” *Ocular Immunology and Inflammation*, vol. 31, no. 1. 2023. doi: 10.1080/09273948.2021.1995763.

[3] A. Baharani and R. R. Reddy, “Multiple Evanescent White Dot Syndrome Following Adenovirus Vector-Based COVID-19 Vaccine (Covishield),” *Ocular Immunology and Inflammation*. 2023. doi: 10.1080/09273948.2023.2192271.

[4] T. R. Beketova, K. Snyder, A. Jiang, and R. G. Josephberg, “Acute Posterior Multifocal Placoid Pigment Epitheliopathy With Associated Papillitis,” *Cureus*, 2023, doi: 10.7759/cureus.35499.

[5] S. Bellur, A. Zeleny, M. Patronas, K. Jiramongkolchai, and S. Kodati, “Bilateral Acute Macular Neuroretinopathy after COVID-19 Vaccination and Infection,” *Ocular Immunology and Inflammation*. 2022. doi: 10.1080/09273948.2022.2093753.

[6] A. D. Bøhler, M. E. Strøm, K. U. Sandvig, M. C. Moe, and Ø. K. Jørstad, “Acute macular neuroretinopathy following COVID-19 vaccination,” *Eye 2021 36:3*, vol. 36, no. 3, pp. 644–645, Jun. 2021, doi: 10.1038/s41433-021-01610-1.

[7] B. A. J. Book, B. Schmidt, and A. M. H. Foerster, “Bilateral Acute Macular Neuroretinopathy after Vaccination against SARS-CoV-2,” *JAMA Ophthalmology*, vol. 139, no. 7. 2021. doi: 10.1001/jamaophthalmol.2021.2471.

[8] C. S. Chean, E. Ali, P. Kulkarni, B. Kapoor, and P. Kumar, “Bilateral Persistent Placoid Maculopathy Following COVID-19 Vaccines: Real or Coincidence?,” *Ocular Immunology and Inflammation*. 2023. doi: 10.1080/09273948.2023.2170889.

[9] D. Drüke, U. Pleyer, H. Hoerauf, N. Feltgen, and S. Bemme, “Acute macular neuroretinopathy (AMN) following COVID-19 vaccination,” *Am J Ophthalmol Case Rep*, vol. 24, 2021, doi: 10.1016/j.ajoc.2021.101207.

[10] S. Fekri, M. Khorshidifar, M. S. Dehghani, H. Nouri, and S. H. Abtahi, “Acute macular neuroretinopathy and COVID-19 vaccination: Case report and literature review,” *J Fr Ophtalmol*, vol. 46, no. 1, pp. 72–82, Jan. 2023, doi: 10.1016/J.JFO.2022.09.008.

[11] P. H. Gabrielle *et al.*, “Bilateral acute macular neuroretinopathy in a young woman after the first dose of Oxford–AstraZeneca COVID-19 vaccine,” *Am J Ophthalmol Case Rep*, vol. 25, 2022, doi: 10.1016/j.ajoc.2022.101281.

[12] M. Goyal, S. I. Murthy, and S. Annum, “Bilateral Multifocal Choroiditis following COVID-19 Vaccination,” *Ocular Immunology and Inflammation*, vol. 29, no. 4. 2021. doi: 10.1080/09273948.2021.1957123.

[13] M. Hébert, S. Couture, and I. Schmit, “Bilateral Panuveitis with Occlusive Vasculitis following Coronavirus Disease 2019 Vaccination,” *Ocular Immunology and Inflammation*, vol. 31, no. 3. 2023. doi: 10.1080/09273948.2022.2042325.

[14] S. Inagawa *et al.*, “Multiple evanescent white dot syndrome following vaccination for COVID-19 A case report,” *Medicine (United States)*, vol. 101, no. 2, 2022, doi: 10.1097/MD.0000000000028582.

[15] N. Jakirlic and T. Harris, “Case Report: Acute Posterior Multifocal Placoid Pigment Epitheliopathy after SARS-CoV-2 Vaccination,” *Optometry and Vision Science*, vol. 99, no. 6, 2022, doi: 10.1097/OPX.0000000000001900.

[16] C. Lee *et al.*, “Neuroretinitis after the second injection of a SARS-CoV-2-vaccine: A case report,” *Am J Ophthalmol Case Rep*, vol. 27, 2022, doi: 10.1016/j.ajoc.2022.101592.

[17] K. S. Lin and M. H. Hsieh, “Multiple Evanescent White Dot Syndrome Following Medigen Vaccine Biologics Corporation COVID-19 Vaccination,” *Ocular Immunology and Inflammation*, vol. 30, no. 5. 2022. doi: 10.1080/09273948.2022.2062388.

[18] K. McElhinney, R. McGrath, E. Ahern, and E. O’Connell, “Bilateral acute posterior multifocal placoid pigment epitheliopathy (APMPPE) following SARS-CoV-2 mRNA vaccine,” *BMJ Case Reports*, vol. 15, no. 6. 2022. doi: 10.1136/bcr-2022-250346.

[19] T. Michel *et al.*, “Acute macular neuroretinopathy after COVID-19 vaccine,” *J Fr Ophtalmol*, vol. 45, no. 7, pp. e299–e302, Sep. 2022, doi: 10.1016/J.JFO.2022.01.022.

[20] G. S. Nair, I. A. Khan, S. W. A. Rizvi, and S. Shahid, “A Case of Neuroretinitis following Inactivated Virion COVID-19 Vaccination,” *Ocular Immunology and Inflammation*. 2023. doi: 10.1080/09273948.2023.2173244.

[21] L. Ninet, A. Comet, D. Denis, and T. David, “Multiple Evanescent White Dot Syndrome following BioNTech SARS-CoV2 mRNA vaccination,” *Journal Francais d’Ophtalmologie*, vol. 45, no. 7. 2022. doi: 10.1016/j.jfo.2022.03.002.

[22] Y. Ogino *et al.*, “A case of APMPPE-like panuveitis presenting with extensive outer retinal layer impairment following COVID-19 vaccination,” *BMC Ophthalmol*, vol. 23, no. 1, 2023, doi: 10.1186/s12886-023-02978-2.

[23] S. N. Patel and Y. Yonekawa, “ACUTE MACULAR NEURORETINOPATHY after SARS-COV-2 VACCINATION,” *Retin Cases Brief Rep*, vol. 16, no. 1, pp. 5–8, Jan. 2022, doi: 10.1097/ICB.0000000000001195.

[24] F. Pichi, S. Aljneibi, P. Neri, S. Hay, C. Dackiw, and N. G. Ghazi, “Association of Ocular Adverse Events with Inactivated COVID-19 Vaccination in Patients in Abu Dhabi,” *JAMA Ophthalmol*, vol. 139, no. 10, 2021, doi: 10.1001/jamaophthalmol.2021.3477.

[25] M. Abrishami, S. Hosseini, N. Shoeibi, and H. Heidarzadeh, “Unilateral acute central serous chorioretinopathy with inactivated coronavirus disease 2019 vaccination: A case report and review of literature,” *J Curr Ophthalmol*, vol. 34, no. 3, 2022, doi: 10.4103/joco.joco_41_22.

[26] A. T. Rennie, A. J. DeWeerd, M. G. Martinez, and C. N. Kay, “Acute Macular Neuroretinopathy Following COVID-19 mRNA Vaccination,” *Cureus*, 2022, doi: 10.7759/cureus.27502.

[27] S. Sanjay *et al.*, “Bilateral Sequential Acute Macular Neuroretinopathy in an Asian Indian Female with β Thalassemia Trait following (Corona Virus Disease) COVID-19 Vaccination and Probable Recent COVID Infection - Multimodal Imaging Study,” *Ocular Immunology and Inflammation*, vol. 30, no. 5. 2022. doi: 10.1080/09273948.2022.2026978.

[28] H. J. Seong and C. S. Lee, “Multiple Evanescent White Dot Syndrome with Submacular Fluid in Dome-shaped Macula Following COVID-19 Vaccination: A Case Report,” *Korean Journal of Ophthalmology*, vol. 36, no. 5. 2022. doi: 10.3341/kjo.2022.0077.

[29] M. Soifer, N. V. Nguyen, R. Leite, J. Fernandes, and S. Kodati, “Recurrent Multiple Evanescent White Dot Syndrome (MEWDS) Following First Dose and Booster of the mRNA-1273 COVID-19 Vaccine: Case Report and Review of Literature,” *Vaccines (Basel)*, vol. 10, no. 11, 2022, doi: 10.3390/vaccines10111776.

[30] K. S. Tomishige, E. A. Novais, L. P. Luciana, H. M. do Nascimento, and R. Belfort, “Multiple evanescent white dot syndrome (MEWDS) following inactivated COVID-19 vaccination (Sinovac-CoronaVac),” *Arquivos Brasileiros de Oftalmologia*, vol. 85, no. 2. 2022. doi: 10.5935/0004-2749.20220070.

[31] D. A. Valenzuela, S. Groth, K. J. Taubenslag, and S. Gangaputra, “Acute macular neuroretinopathy following Pfizer-BioNTech COVID-19 vaccination,” *Am J Ophthalmol Case Rep*, vol. 24, Dec. 2021, doi: 10.1016/J.AJOC.2021.101200.

[32] S. Vinzamuri, T. G. Pradeep, and R. Kotian, “Bilateral paracentral acute middle maculopathy and acute macular neuroretinopathy following COVID-19 vaccination,” *Indian J Ophthalmol*, vol. 69, no. 10, pp. 2862–2864, Oct. 2021, doi: 10.4103/IJO.IJO_1333_21.

[33] Z. C. Wiley, M. Pakravan, C. Charoenkijkajorn, S. C. Kavoussi, and A. G. Lee, “Uveomeningeal syndrome presenting with bilateral optic disc edema and multiple evanescent white dots syndrome (MEWDS),” *Am J Ophthalmol Case Rep*, vol. 26, 2022, doi: 10.1016/j.ajoc.2022.101538.

[34] Y. Xu and W. Shen, “Presumed Recurrent MEWDS following Covid-19 Vaccination,” *Ocular Immunology and Inflammation*, vol. 29, no. 6. 2021. doi: 10.1080/09273948.2021.1985524.

[35] E. Yasuda *et al.*, “Multiple evanescent white dot syndrome following BNT162b2 mRNA COVID-19 vaccination,” *Am J Ophthalmol Case Rep*, vol. 26, 2022, doi: 10.1016/j.ajoc.2022.101532.

[36] N. Zaheer, M. P. Renju, and R. Chavan, “ACUTE MACULAR NEURORETINOPATHY AFTER COVID-19 VACCINATION,” *Retin Cases Brief Rep*, vol. 16, no. 1, pp. 9–11, Jan. 2022, doi: 10.1097/ICB.0000000000001196.

[37] A. Arora *et al.*, “Recurrence of tubercular choroiditis following anti-SARS-CoV-2 vaccination,” *Eur J Ophthalmol*, vol. 33, no. 3, 2023, doi: 10.1177/11206721221088439.

[38] E. Bolletta *et al.*, “Uveitis and other ocular complications following covid-19 vaccination,” *J Clin Med*, vol. 10, no. 24, 2021, doi: 10.3390/jcm10245960.

[39] S. Bouhout, M. Hébert, J. Vadboncoeur, and M. J. Aubin, “Multiple evanescent white dot syndrome following COVID-19 vaccines,” *Canadian Journal of Ophthalmology*, vol. 58, no. 3. 2023. doi: 10.1016/j.jcjo.2022.10.002.

[40] A. Franchi *et al.*, “Two Cases of Acute Macular Neuroretinopathy Associated with the Adenovirus-based COVID-19 Vaccine Vaxzevria (Astrazeneca),” *Ocular Immunology and Inflammation*, vol. 30, no. 5. 2022. doi: 10.1080/09273948.2022.2027463.

[41] M. A. Gargouri *et al.*, “Multiple Evanescent White Dot Syndrome Following COVID-19 mRNA Vaccination,” *Ocular Immunology and Inflammation*. 2022. doi: 10.1080/09273948.2022.2127782.

[42] C. Girbardt *et al.*, “Retinal vascular events after mrna and adenoviral-vectored covid-19 vaccines—a case series,” *Vaccines (Basel)*, vol. 9, no. 11, 2021, doi: 10.3390/vaccines9111349.

[43] K. Ishibashi, H. Yatsuka, M. Haruta, K. Kimoto, S. Yoshida, and T. Kubota, “Branch Retinal Artery Occlusions, Paracentral Acute Middle Maculopathy and Acute Macular Neuroretinopathy After COVID-19 Vaccinations,” *Clinical Ophthalmology*, vol. 16, 2022, doi: 10.2147/OPTH.S357359.

[44] M. B. Jalink and I. H. G. Bronkhorst, “A Sudden Rise of Patients with Acute Macular Neuroretinopathy during the COVID-19 Pandemic,” *Case Rep Ophthalmol*, 2022, doi: 10.1159/000522080.

[45] S. Li *et al.*, “Intraocular inflammation following COVID-19 vaccination: the clinical presentations,” *Int Ophthalmol*, vol. 43, no. 8, pp. 2971–2981, Aug. 2023, doi: 10.1007/S10792-023-02684-4/TABLES/2.

[46] Z. Li *et al.*, “Ocular Adverse Events after Inactivated COVID-19 Vaccination,” *Vaccines (Basel)*, vol. 10, no. 6, 2022, doi: 10.3390/vaccines10060918.

[47] M. Mambretti, J. Huemer, G. Torregrossa, M. Ullrich, O. Findl, and G. Casalino, “Acute Macular Neuroretinopathy following Coronavirus Disease 2019 Vaccination,” *Ocular Immunology and Inflammation*, vol. 29, no. 4. 2021. doi: 10.1080/09273948.2021.1946567.

[48] T. Rabinovitch *et al.*, “Uveitis following the BNT162b2 mRNA vaccination against SARS-CoV-2 infection: a possible association.,” *Retina*, 2021, doi: 10.1097/IAE.0000000000003277.

[49] E. Smith, T. Tran, A. Gillies, S. Yeung, and P. E. Ma, “Multiple Evanescent White Dot Syndrome following COVID-19 mRNA Vaccination in Two Patients,” *Ocular Immunology and Inflammation*, vol. 30, no. 5. 2022. doi: 10.1080/09273948.2022.2032198.

[50] L. U. Wang *et al.*, “Ocular inflammatory manifestations following COVID-19 vaccinations in Taiwan: A case series,” *Taiwan J Ophthalmol*, vol. 12, no. 4, 2022, doi: 10.4103/2211-5056.353129.
